# Massive Amplification at an Unselected Locus Accompanies Complex Chromosomal Rearrangements in Yeast

**DOI:** 10.1534/g3.115.024547

**Published:** 2016-03-04

**Authors:** Agnès Thierry, Varun Khanna, Bernard Dujon

**Affiliations:** Institut Pasteur, Unité de Génétique Moléculaire des Levures, CNRS (UMR3525), Sorbonne Universités, UPMC, Université Paris 06 (UFR927), F-75724 CEDEX 15, France

**Keywords:** tRNA synthetase, *CUP1* amplification, macrotene chromosomes, quasi-palindromes, disomy

## Abstract

Gene amplification has been observed in different organisms in response to environmental constraints, such as limited nutrients or exposure to a variety of toxic compounds, conferring them with specific phenotypic adaptations via increased expression levels. However, the presence of multiple gene copies in natural genomes has generally not been found in the absence of specific functional selection. Here, we show that the massive amplification of a chromosomal locus (up to 880 copies per cell) occurs in the absence of any direct selection, and is associated with low-order amplifications of flanking segments in complex chromosomal alterations. These results were obtained from mutants with restored phenotypes that spontaneously appeared from genetically engineered strains of the yeast *Saccharomyces cerevisiae* suffering from severe fitness reduction. Grossly extended chromosomes (macrotene) were formed, with complex structural alterations but sufficient stability to propagate unchanged over successive generations. Their detailed molecular analysis, including complete genome sequencing, identification of sequence breakpoints, and comparisons between mutants, revealed novel mechanisms causing their formation, whose combined action underlies the astonishing dynamics of eukaryotic chromosomes and their consequences.

Structural changes and copy number variations are frequently observed alterations of eukaryotic chromosomes, with potentially important evolutionary consequences as well as possible deleterious effects that are able to determine pathological processes in humans (Kloosterman *et al.* 2015; Zarrei *et al.* 2015). In the yeast *Saccharomyces cerevisiae*, low-order amplifications (mostly duplications) of large chromosomal segments are often observed in evolutionary experiments aimed at studying adaptation to limiting environmental conditions (Dunham *et al.* 2002; Gresham *et al.* 2008, 2010; Araya *et al.* 2010; Payen *et al.* 2014) or the recovery from artificial gene dosage imbalance (Koszul *et al.* 2004; Libuda and Winston 2006; Payen *et al.* 2008). These events generate a variety of topological forms, including intra- or interchromosomal segmental duplications, the formation of additional chromosomes (neochromosomes made by the junction of two or more duplicated segments), or independent episomes in circular or linear forms. Higher order gene amplifications were also observed in cultivated mammalian cells exposed to methotrexate (Alt *et al.* 1978) or in plants exposed to glyphosate (Gaines *et al.* 2010) with, in the latter case, multiple copies dispersed on the different chromosomes. In *S. cerevisiae*, expansions of tandem gene arrays were observed long ago at the *CUP1* locus on chromosome VIII in response to exposure to toxic copper salts (Welch *et al.* 1983; Karin *et al.* 1984) and, more recently, at the *HXT6-HXT7* locus on chromosome IV in response to glucose limitation (Brown *et al.* 1998). If dozens of short tandem arrays (typically two to three gene copies) scatter normal genomes of all yeast species (Dujon *et al.* 2004; Despons *et al.* 2010, 2011), larger arrays are also occasionally encountered in natural yeast genomes, often showing polymorphic size variation in populations as illustrated, for example, by the *ENA1-ENA2-ENA5* locus encoding a P-type ATPase sodium pump in *S. cerevisiae* (Martinez *et al.* 1991; Wieland *et al.* 1995) or by genes encoding α-1,3-mannosyl transferases in the pathogenic yeast *Candida glabrata* (Muller *et al.* 2009).

In addition to these phenomena, high order amplifications of large chromosomal segments, leading to significant chromosome size expansions (macrotene chromosomes), were recently discovered in phenotypically restored revertants from genetically engineered *S. cerevisiae* strains (Thierry *et al.* 2015). In these strains, the replacement of essential tRNA synthetase genes (RS genes) by their orthologs from another, distantly related yeast species (*Yarrowia lipolytica*) resulted in severe fitness reduction due the insufficient charge of the pools of tRNA molecules by their cognate amino acids. Experimental evidence indicated that the multiple *in loco* repeats formed in macrotene chromosomes were the result of single-step massive accidental events during DNA replication, rather than selection-driven, successive illegitimate recombination between copies. In humans, complex genomic rearrangements resulting from single catastrophic events, designated chromothripsis, were also recently identified in cancer cells or in cells of patients suffering from congenital developmental disorders (Stephens *et al.* 2011; Kloosterman and Cuppen, 2013; Korbel and Campbell 2013; Zhang *et al.* 2013). In such cases, pieces from a shattered chromosome arm are stitched together in apparently random order, orientation, and number, producing deletions and duplications. However, to our knowledge, no direct relationship has been reported so far between large chromosomal rearrangements associated with moderate copy number variation and massive, selection-driven gene amplifications. The results presented here establish such a relationship and suggest a mechanism for the formation of such grossly altered chromosomal structures.

Using our previously constructed transgenic *S. cerevisiae* strains (Thierry *et al.* 2015), in which the essential Asn (asparagine) tRNA synthetase gene (*DED81*) was replaced by its ortholog from *Y. lipolytica*, we now report the spontaneous formation of novel chromosomal structures bearing massive amplifications of the 2 kb-long *CUP1* locus (up to over 800 copies per cell), associated with complex low-order amplifications and rearrangements of large chromosomal segments affecting one or two distinct chromosome(s). Contrary to previous reports (Welch *et al.* 1983; Adamo *et al.* 2012; Chang *et al.* 2013; Zhao *et al.* 2014), the amplification of the *CUP1* locus in our mutants is not only much greater (by at least one order of magnitude) but is also not selected for, based on copper resistance. The concomitant formation of massively amplified *CUP1* loci with the low-order amplifications of large chromosomal segments, which include the Asn-RS transgene on which the phenotypic selection is based, illustrates how catastrophic alterations occur in normal eukaryotic chromosomes. We report here the detailed analysis of such events and propose underlying mechanisms based on genome comparisons between different mutants in two independent evolutionary experiments.

## Materials and Methods

### Culture conditions

Yeast strains were grown on YPD medium (Yeast extract 10 g/L, Bacto peptone 10 g/L, glucose 20 g/L, with or without 25 g/L bacto-agar as needed) at 30°, unless otherwise indicated.

### Sporulation and parental strain construction

Tetrads from diploid strains BYAT580-0 and BYAT580-200 (Thierry *et al.* 2015) were micromanipulated using Singer MSM equipment. Ascospores were inoculated on thin YPD agar medium and incubated at 30° for 3 days, following which the agar was placed on top of new YPD plates to ensure sufficient nutrient availability for slow-growing strains, and further incubated for several days (see Supplemental Material, Figure S1). Selected haploid segregants were immediately grown in patches on YPD medium for a limited number of generations. Cells were stored at –80° (see File S1).

### Pulsed-field gel electrophoresis (PFGE)

**Figure 1:**
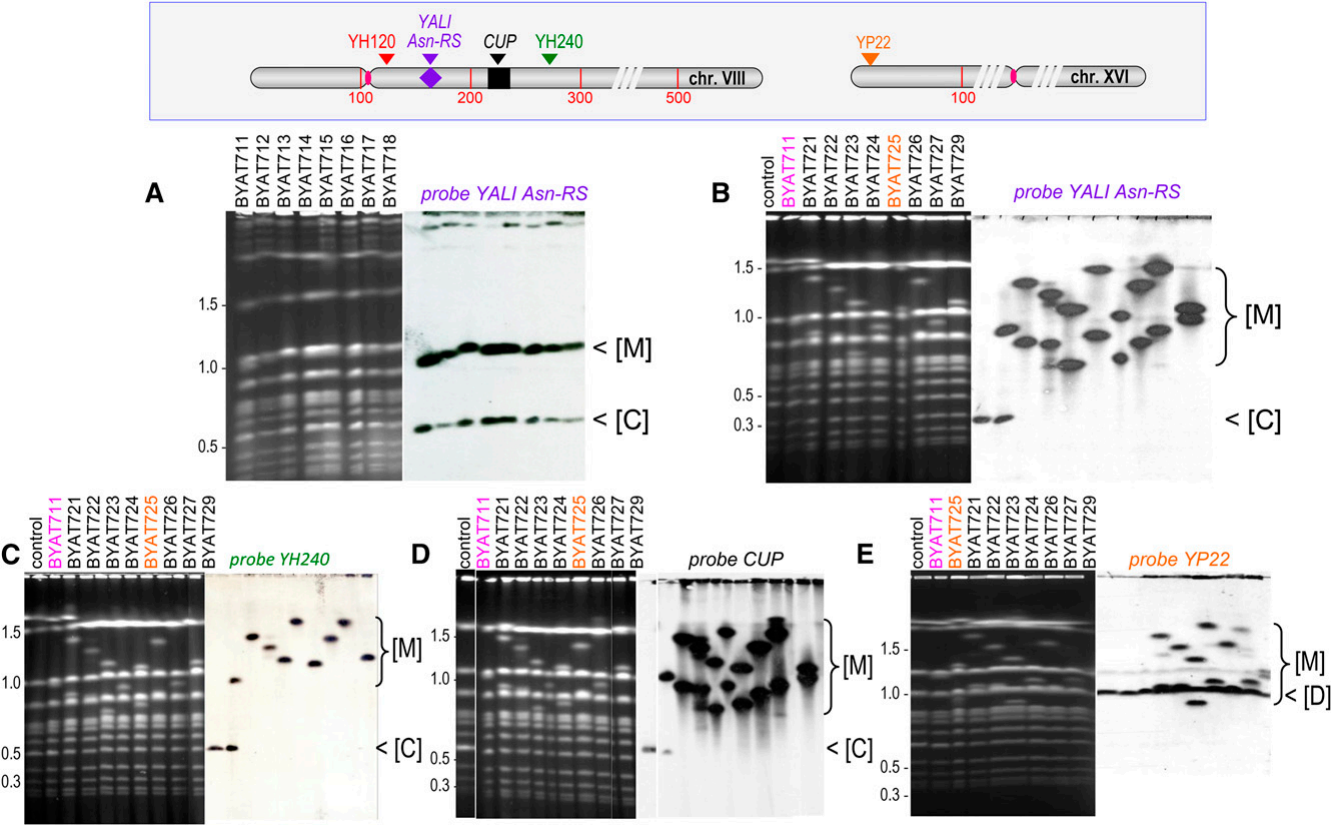
Pulsed-field gel electrophoresis (PFGE) of evolved mutants. Electrophoresis was conducted as described in *Materials and Methods*. Left: ethidium bromide fluorescence, size scales in Mb calibrated from migration of natural *S. cerevisiae* chromosomes. Right: hybridization with indicated probes. (A) Evolved mutants from strain BYAT3C. (B–E) Evolved mutants from strain BYAT8A (plus parental strain BYAT521 and mutant BYAT711 used as controls). Note that BYAT722 was probably a mixed colony. (Top box) Approximate positions of probes along chromosomes VIII and XVI (coordinates in kb, see Table S3 for details). [C], normal chromosome VIII; [D], normal chromosome XVI; [M], macrotene chromosomes.

Blocks of native chromosomes were prepared from agarose-embedded yeast cells according to standard methods (Schwartz and Cantor 1984). PFGE was run on 1% agarose gels, in 0.25 × TBE buffer at pH 8.3 at 12°, and 5 V/cm for 65 hr on a Rotaphor (Biometra) with an alternating field angle of 120° and pulse ramps of 300–100 sec (Figure 1A) or 180–80 sec (Figure 1, B–E, Figure 6, and Figure 7), with linear accelerations. PFGE of *Eco*RI or *Bam*HI digests were run on 1% agarose gels, in 0.25 × TBE buffer at pH 8.3 at 12°, and 6 V/cm for 30 hr on a Rotaphor (Biometra) with an alternating field angle of 110–95° and a pulse ramp of 100–20 sec with logarithmic acceleration (Figure 3). Southern blots of PFGE were hybridized using digoxigenin-labeled probes, according to the manufacturer’s protocol (Roche, https://lifescience.roche.com/shop/en/fr/products/detection-of-dig-labeled-probes).

### Evolutionary experiments

Subclones from parental strains were inoculated in YPD liquid medium and grown at 30° by serial transfer for a total of ∼30–35 successive generations (see Figure S1). Each transfer was made in 2 L fresh medium with inoculums of 10^9^ cells. Under such conditions, each culture represented ∼8 generations (Thierry *et al.* 2015). After each culture, cells were diluted and plated on YPD solid medium to monitor the growth and morphology of the resulting colonies. Faster growing mutants (larger colonies) were selected for molecular analysis.

### DNA sequencing

Total DNA purified from yeast cultures was fragmented using a Bioruptor (Diagenode) and libraries were prepared using the TruSeq DNA sample preparation method (Illumina, TruSeq cluster SR v3 and TruSequation 50 cycles SBS v3). Sequences were read on HiSeq2000 (Illumina) at high coverage (384–834 ×).

### Sequence analysis

**Figure 2:**
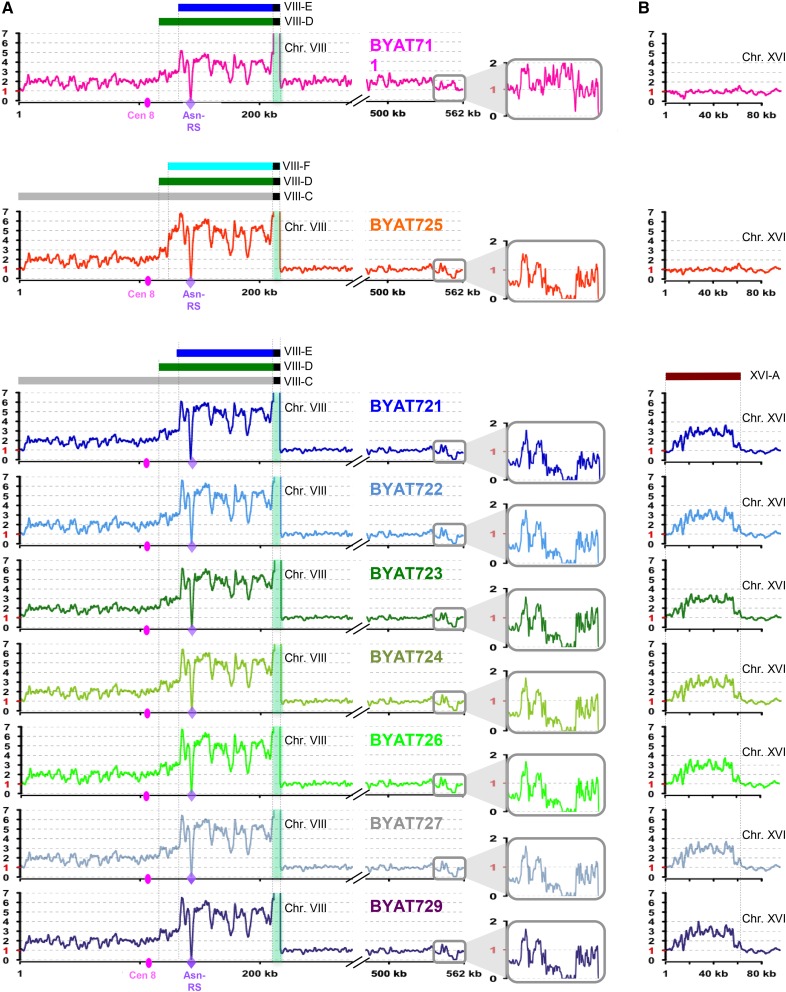
Amplicons identified from genome sequencing coverages. (A) Copy number variation along chromosome VIII, as determined from sequence coverage of evolved mutants (see *Materials and Methods*). For mutant numbering, refer to Table S1. Copy numbers (ordinates) were normalized to 1 (for haploid) using median coverage of other chromosomes (see Figure S2). Curves were generated using R version 3.1.2 (http://www.R-project.org) and smoothed over 1500 nucleotide sliding windows (fluctuations correlate with GC %). (B) Copy number variation along the left-end segment of chromosome XVI. Horizontal colored bars symbolize amplicons (see text and Figure 5). Abrupt curve drops correspond to the *Y. lipolytica* Asn-RS gene (purple diamond) absent from the *S. cerevisiae* reference sequence. Pink oval: centromere. Light green background highlights the *CUP1* locus (cut for drawing clarity, see Figure 3). Central windows zoom to the 13 kb-long right subtelomeric segment of chromosome VIII beyond coordinate 540 kb (curves smoothed using 200 nucleotide sliding windows, see text for interpretation). Asn-RS, asparagine tRNA synthetase; Chr, chromosome.

**Figure 3:**
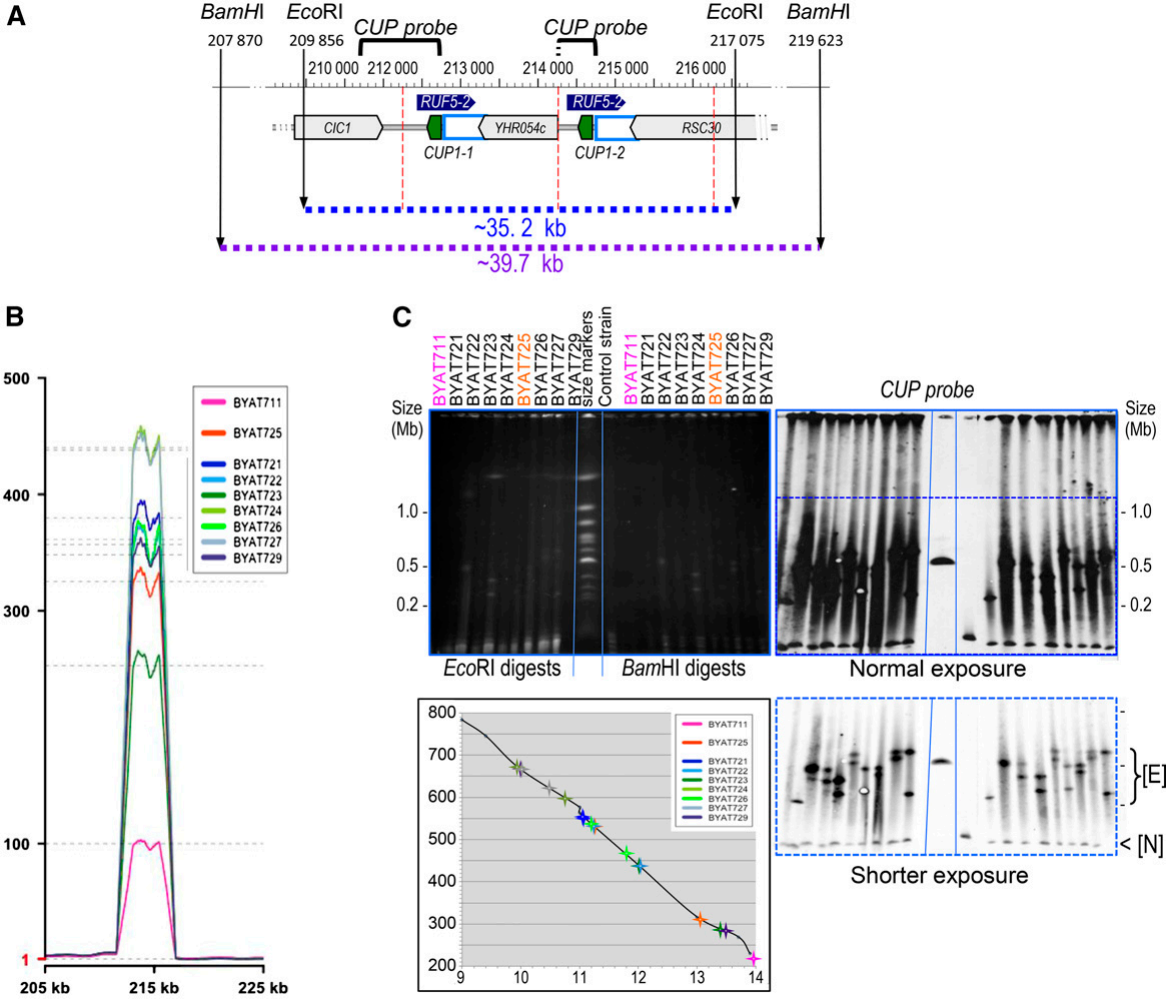
Analysis of the CUP amplifications. (A) Map of the *CUP1* locus on chromosome VIII deduced from the reference S288c sequence. The 2 kb repeat units are delimited by the vertical dotted red lines. Gray arrowhead boxes: protein-coding genes, void light blue boxes: replication origins (ARS810). The *CUP1* genes are colored in green and the antisense transcripts *RUF5-2* are materialized by the dark blue arrows. Sizes indicated for *Eco*RI and *Bam*HI restriction fragments correspond to the parental strains used in this work, containing 16 copies of the repeat unit. (B) Copy number variation over the *CUP1* locus, as determined from sequence coverage of evolved mutants. Same legend as Figure 2. (C) Pulsed-field gel electrophoresis (see *Materials and Methods*) of restriction digests of total DNA from evolved mutants. Top left: ethidium bromide fluorescence, size scale in Mb calibrated from migration of normal *S. cerevisiae* chromosomes (size markers lane). Right: hybridization with the CUP probe (below: shorter exposure of selected area to facilitate measurements of hybridizing bands, size marks repeated on right). Control strain: BYAT521. [N]: restriction fragment of normal *CUP1* locus; [E] restriction fragments of expanded *CUP1* loci. Bottom left insert: migration of hybridizing bands of evolved mutants was reported on the calibration curve (abscissae: migration in cm, ordinate: size in kb) for precise size determination (see Table 2).

Bioinformatics analysis followed the pipeline illustrated by Figure S5. Regions of problematic base calling (deviation from Chargaff’s rules) were visualized by FastQC v0.10.1 (Babraham Bioinformatics, http://www.bioinformatics.babraham.ac.uk/projects/fastqc/) and trimmed off using Trimmomatic v0.30 (Bolger *et al.* 2014). Trimmed reads were then aligned against reference sequences of the 16 chromosomes plus the mitochondrial DNA of *S. cerevisiae* S288c (Genbank NC_001133 to NC_001148, GenBank NC_001224, PLN 06-DEC-2008) and the Asn-RS gene of *Y. lipolytica* (*YALI0E05005g*), using the single end mapping mode of BWA v0.6.2 (Li and Durbin 2009) with default parameters. Output SAM files were converted to BAM files using SAMtools v0.1.18 (Li *et al.* 2009). Sequencing coverage at each position was computed by BEDtools v2.17.0 (Quinlan and Hall 2010) and compared to median coverage over the entire genome (normalized to 1 for haploid cells) to calculate copy number per cell. When necessary, coverages were smoothed using 1500 nucleotide (nt) (Figure 2 and Figure 3) or 5000 nt sliding windows (Figure S2). Copy number plots were drawn using R version 3.1.2 (http://www.R-project.org/). Tablet (Milne *et al.* 2013) and IGV (Robinson *et al.*, 2011) were used to visualize alignments between the BAM files and reference sequences and to screen for the possible presence of nucleotide substitutions and indels at critical loci (see *Discussion*). In order to identify novel junction sequences corresponding to breakpoints, all unmapped reads against the reference sequence (extracted using SAMtools v0.1.18 with the option “view -f 4”) were compiled into a SAM file, converted to a Fastq file using the Picard v1.81 “SamToFastq.jar” tool (http://picard.sourceforge.net/), and then submitted to reassembly using Spades 3.0.0 (Nurk *et al.* 2013) with the kmer option “-k 21,51,71.” The resulting contigs with sufficient sequence coverage (corresponding to > 0.5 copy per genome) were manually inspected to identify possible breakpoints against the reference sequence.

### Data availability

Strains are available upon request (athierry@pasteur.fr). DNA sequence data for BYAT711, BYAT725, BYAT721, BYAT722, BYAT723, BYAT724, BYAT726, BYAT727, and BYAT729 have been deposited in the European Nucleotide Archive (http://www.ebi.ac.uk/ena/data/view/PRJEB8937) under the accession codes ERS688167, ERS688168, ERS688169, ERS688170, ERS688171, ERS688172, ERS688173, ERS688174, and ERS688175, respectively.

## Results

### Extensive chromosomal size variation and disomy in evolved mutants

The delicate construction of the transgenic parental strains used in this work and the selection of evolved mutants are detailed in File S1 and summarized by Figure S1. In brief, two haploid strains, *BYAT3C* and *BYAT8A*, in which the essential Asn tRNA synthetase gene (*DED81*) on chromosome VIII was replaced by its ortholog *YALI0E05005g* from *Y. lipolytica* (see Table S1), were used to start the experiments after having verified their normal genomic structures (see Table S2). The two strains were meiotic segregants of previously characterized diploid strains (Thierry *et al.* 2015) and exhibited extremely reduced growth rates on rich glucose medium, as expected. They were immediately stored at –80° after the smallest possible number of generations after meiosis to prevent the accumulation of undesirable mutants before the start of the experiments. Evolution experiments were carried out by serial transfer in rich glucose medium from subclones of each parental strain (see Figure S1). Eight evolved mutants were isolated from each experiment by picking the largest colonies among the heterogeneous populations that rapidly formed during cultivation. Table S1 lists the parental strains and evolved mutants analyzed in this work, with their origin and genotype.

Evolved mutants were first analyzed by PFGE, showing surprising results (Figure 1). Instead of the normal chromosome VIII migrating at its 563 kb size (as observed in the control), each mutant showed two bands hybridizing with the *YALI*-Asn-RS probe; an unexpected result given the fact that the original strains were haploid and that all mutants remained haploid (see below). Furthermore, the two evolutionary experiments gave strikingly different results. All eight mutants from strain *BYAT3C* showed a similar pattern, as if an earlier mutational event had propagated unchanged in the entire population (Figure 1A). Their shorter band corresponded in size to the normal chromosome VIII, whereas their longer band (note the more intense hybridization) migrated at ∼1090 kb, *i.e.*, nearly twice the size of the normal chromosome VIII, a figure reminiscent of the macrotene chromosomes previously described (Thierry *et al.* 2015). Mutant BYAT711 was selected for all subsequent analyses (see below), assuming that the other mutants were identical. In contrast, the eight mutants from strain *BYAT8A* showed extensive diversity, and none were similar to the first set of mutants (Figure 1B). They all lost the normal size chromosome VIII and, instead, exhibited two larger bands (again reminiscent of macrotene chromosomes) hybridizing with the *YALI*-Asn-RS probe (note the intense hybridization in both cases), ranging in size from 890–1530 kb, *i.e.*, 1.6–2.7 times the size of the normal chromosome VIII (for precise sizes, refer to Table 1).

**Table 1:** Size of hybridizing bands of undigested chromosomal DNA on pulsed-field gel electrophoresis

Evolved Mutant	*YALI* Asn-RS	YH240	YP22	*CUP*
BYAT711	**563***a*	**563***a*	—	**563***a*
	1090	1090	—	1090
BYAT725	890	—	—	890
	1130	1130	—	1130
BYAT721	990	—	990	990
	1400	1400	1400	1400
BYAT722	990	—	990	990*b*
	1300	1300	1300	1300*b*
BYAT723	890	—	890	890
	1150	1150	1150	1150
BYAT724	1030	—	1030	1030
	1530	1530	1530	1530
BYAT726	990	—	990	990
	1350	1350	1350	1350
BYAT727	1090	—	1090	1090*b*
	1530	1530	1530	1530*b*
BYAT729	1000	—	1000	1000
	1150	1150	1150	1150

For each strain (left column), the table indicates the size in kb of the chromosomal band(s) hybridizing, respectively, with the probes indicated on top. Data measured from Figure 1 using native yeast chromosomes for calibration. Normal size chromosome VIII is shown in bold. Absence of hybridization with a specific probe is indicated by –.

a Size of chromosome VIII in the reference S288c sequence includes two copies of the *CUP1* repeat unit.

b Smear or multiple bands.

As a first attempt to identify the nature of these novel macrotene chromosomes, we used a probe (YH240) marking the right arm of chromosome VIII at coordinates 239–240 kb (see Table S3). The results, shown in Figure 1C, indicated similar hybridization intensities for the normal size chromosome VIII and the macrotene chromosome in BYAT711, suggesting disomy. In contrast, for all mutants derived from *BYAT8A*, only the larger of the two macrotene chromosomes hybridized with the probe, indicating that, although larger than normal, the smaller one did not contain the entire chromosome VIII.

### Amplifications of chromosomal segments deduced from whole-genome sequences

To examine the unexpected genomic structures of our evolved mutants, we sequenced each of them at high coverage and analyzed the sequences as indicated in *Materials and Methods*. When mapped against the reference S288c genome, sequencing coverage proved homogenous over entire genomes, indicating an absence of copy number alteration except for chromosome VIII (see Figure S2). In addition, a short segment of chromosome XVI corresponding to the left subtelomeric regions was also amplified in seven of the eight mutants of *BYAT8A* (BYAT725 remained normal). Therefore, we concluded that our mutants remained haploid, like their parental strains, but exhibited segmental amplifications. Only mutant BYAT711 showed complete chromosome VIII disomy, as judged from double sequencing coverage of its right arm beyond the *CUP1* locus (consistent with the hybridization of both bands with probe YH240, see Figure 1C). Detailed examination of the amplified regions along chromosomes VIII and XVI revealed a set of interesting amplifications, out of which several amplicons could be precisely defined (Figure 2).

For mutant BYAT711, in addition to disomy, the sequencing coverage over chromosome VIII further increased in the segment between the centromere and the *CUP1* locus (for the *CUP1* locus, see next paragraph). Detailed examination of sequence reads in this segment using Tablet (Milne *et al.* 2013) suggested the presence of two partially overlapping amplicons (VIII-D and VIII-E), each duplicated, thus raising total copy numbers to three (coordinates 116.5–133.8 kb) and then four (from coordinate 133.8 kb to the left side of the *CUP1* locus). These amplicons are large (93.5 kb and 76 kb for VIII-D and VIII-E, respectively), and include the Asn-RS locus among numerous flanking genes. The copy number increase from one to four of the *Y. lipolytica* RS gene is sufficient to explain the phenotypic recovery of mutant BYAT711 compared to its parent (as previously determined in similar experiments by Thierry *et al.* 2015).

In contrast, for all mutants derived from *BYAT8A*, the right part of chromosome VIII after the *CUP1* locus remained haploid (again consistent with hybridizations using the YH240 probe, see Figure 1C), whereas the left arm showed double sequence coverage and the interval between the centromere and the *CUP1* locus was further amplified by the presence of extra copies of amplicons VIII-D and VIII-E, as above (except for strain BYAT725, where the latter was replaced by amplicon VIII-F with a distinct left boundary, see below). Precise amplicon limits were again defined by Tablet analysis. Additional copies of these amplicons raised copy numbers to three (coordinates 116.5–133.8 kb, as above) and five for the segment between coordinate 133.8 kb and the left side of the *CUP1* locus (see next paragraph for the *CUP1* locus). These numbers correspond to two additional copies in excess of haploid number for amplicon VIII-E (VIII-F in the case of BYAT725), and one additional copy in excess of haploid number for amplicons VIII-C and VIII-D. Careful examination of sequence coverage along chromosome VIII in these mutants also revealed that their right 13 kb-long subtelomeric segments after *YHRC*δ*16* (from coordinate 549.5 kb to the telomere) were entirely deleted (central inserts in Figure 2). Note that interpretation of this result was complicated by the fact that an exactly identical sequence (except for the absence of the *IMD2)* exists in the right subtelomeric segment of chromosome I. Consequently, sequence reads originating from chromosome I were erroneously mapped to chromosome VIII, obscuring the deletion (note the coverage lower than one copy and the complete absence of reads corresponding to *IMD2*). Finally, the 56.5 kb-long left subtelomeric region of chromosome XVI was also observed with a total sequencing coverage of three in the seven mutants of this series bearing amplicon VIII-E (but not in BYAT725 bearing amplicon VIII-F), suggesting two additional copies of amplicon XVI-A in excess of haploid number (Figure 2B). Surprisingly, the seven mutants from BYAT8A sharing amplicons VIII-E and XVI-A appeared indistinguishable from one another by sequence coverage, whereas they showed macrotene chromosomes of varying sizes (Figure 1), suggesting that the differences originated from the *CUP1* amplifications.

### High-order multiplication of the CUP amplicon

Sequencing coverage over the *CUP1* locus showed the astonishing result of ∼100 (mutant BYAT711) to ∼450-fold excess (mutants BYAT724 and BYAT727) compared to haploid level (Figure 3). To interpret this result, is it useful to recall that two copies of the 2 kb repeat unit containing the *CUP1* gene and its flanking sequences were arbitrarily included in the reference genome sequence of strain S288c. Our evolved mutants must, therefore, bear ∼200–900 copies of the 2 kb *CUP* amplicon (Table 2) compared to 16 copies for the parental strains (8 × coverage over the two loci of the reference sequence, not shown). The *CUP1* locus was known to vary in repeat number between strains in relation to tolerance to copper salt toxicity (Welch *et al.* 1983; Chang *et al.* 2013; Zhao *et al.* 2014, see *Discussion*) but, to our knowledge, the data obtained here exceed previous numbers by at least one order of magnitude.

**Table 2:** Numbers of *CUP* amplicons in evolved mutants

Evolved Mutant	Size of Hybridizing *Eco*RI Bands	Deduced Number of CUP Amplicons in Chromosome(s)	Total Number of CUP Amplicons in the Cells, Deduced from Sequence Coverage
BYAT711	34.5 + 220	16 + 110 = 126	200
BYAT725	34.5 + 325 + 530	16 + 162 + 265 = 443	650
BYAT721	34.5 + 550 + 560	16 + 275 + 280 = 571	760
BYAT722	34.5 + 450 + 540	16 + 225 + 270 = 511	714
BYAT723	34.5 + 290 + 450	16 + 145 + 225 = 386	506
BYAT724	34.5 + 600 + 690	16 + 300 + 345 = 661	880
BYAT726	34.5 + 470 + 540	16 + 235 + 270 = 521	722
BYAT727	34.5 + 620 + 670	16 + 310 + 335 = 661	876
BYAT729	34.5 + 280 + 675	16 + 140 + 337 = 493	696

For each mutant (left column), the table indicates the size in kb of the *Eco*RI fragments (column 2) hybridizing with the CUP probe (data measured from Figure 3C), the deduced number of CUP amplicons in each fragment, and the total number of CUP repeat units within chromosomes (column 3). For comparison, column 4 indicates the total number of CUP repeat units deduced from sequencing coverages (Figure 3B). Note the systematic difference (1.31–1.59 ratio, see text for explanation).

In order to examine the relationship between the multiplication of the *CUP* amplicon, not directly selected for in our experiments (copper salts were never used), and the low multiplicity of the large amplicons bearing the *YALI* Asn-RS gene selected for in our experiments, we first hybridized the PFGE-separated chromosomes of evolved mutants with a *CUP* probe (Figure 1D). In each mutant from BYAT8A, the two macrotene chromosomes showed high hybridization intensities with the probe, indicating that amplifications of the *CUP1* repeats occurred *in loco* within each altered chromosome VIII copy. For BYAT711, the macrotene chromosome also showed high hybridization intensity with the CUP probe, also indicating *in loco* amplification, whereas the normal-size chromosome VIII showed normal hybridization intensity (comparable to the control), suggesting the absence of amplification.

The absence of *Eco*RI and *Bam*HI sites within the 2 kb *CUP* repeat unit (Figure 3B) offered us a direct method to determine the number of loci containing *CUP* amplicons within the macrotene chromosomes and, for each locus, to directly measure the number of repeats. A normal band migrating at ∼35 kb (*Eco*RI) or ∼40 kb (*Bam*HI) and hybridizing at normal intensity with the probe was observed in each evolved mutant (as well as in the control strain), indicating that the natural *CUP1* locus was preserved in all mutants with the same number of repeats (16) as their parental strains (Figure 3C). However, much larger restriction fragments, with more intense hybridization signals, were also observed in the mutants. Their migration on PFGE indicated sizes ranging from ∼250–670 kb (for precise measurements, see Table 2). For BYAT711, only one such band of ∼250 kb (*Eco*RI) was observed, whereas two such bands, ranging in size from ∼315–670 kb, were present in the other mutants (the two bands comigrated in BYAT721). From these data, we concluded that a single new *CUP1* locus containing ∼124 repeats was present within the *macrotene* chromosome VIII of BYAT711, either in place of the normal *CUP1* locus or in addition to it (the mutant is disomic and it is impossible to tell whether the hybridization level of the normal band corresponds to one or two copies). For the other mutants, we concluded that a single new *CUP1* locus was present in each of the two *macrotene* chromosomes, again either in place of the normal *CUP1* locus or in addition to it (so long as at least one normal locus remained present in order to account for the presence of the normal band). From size measurement of restriction fragments, these new *CUP1* loci must contain from ∼157–335 repeats (Table 2).

It is perhaps interesting to note that the copy numbers of the *CUP1* amplicon, estimated from the *Eco*RI and *Bam*HI digests, were systematically lower (by roughly a third) than those deduced from sequencing coverage. Beside intrinsic technical biases that remain to be quantified, the difference may suggest the presence of extra copies of the CUP amplicon in our mutants, independent from the chromosomes, which may result from an intrinsic instability of the expanded *CUP1* loci in macrotene chromosomes (tandem repeats are bound to loop-out by recombination in *S. cerevisiae*). The presence of hybridizing material in the slots and the important smears observed in both *Eco*RI and *Bam*HI digests (Figure 3C) support these hypotheses. Quantification of the DNA material in the smears from hybridization signals was, however, impossible given the use of nonradioactive probes. Despite this numerical uncertainty about copy number, our evolved mutants have much higher numbers of *CUP1* repeats than any previously described *S. cerevisiae* strains, raising questions about the formation and structure of these novel macrotene chromosomes.

### Novel junctions identified from whole-genome sequences

**Figure 4:**
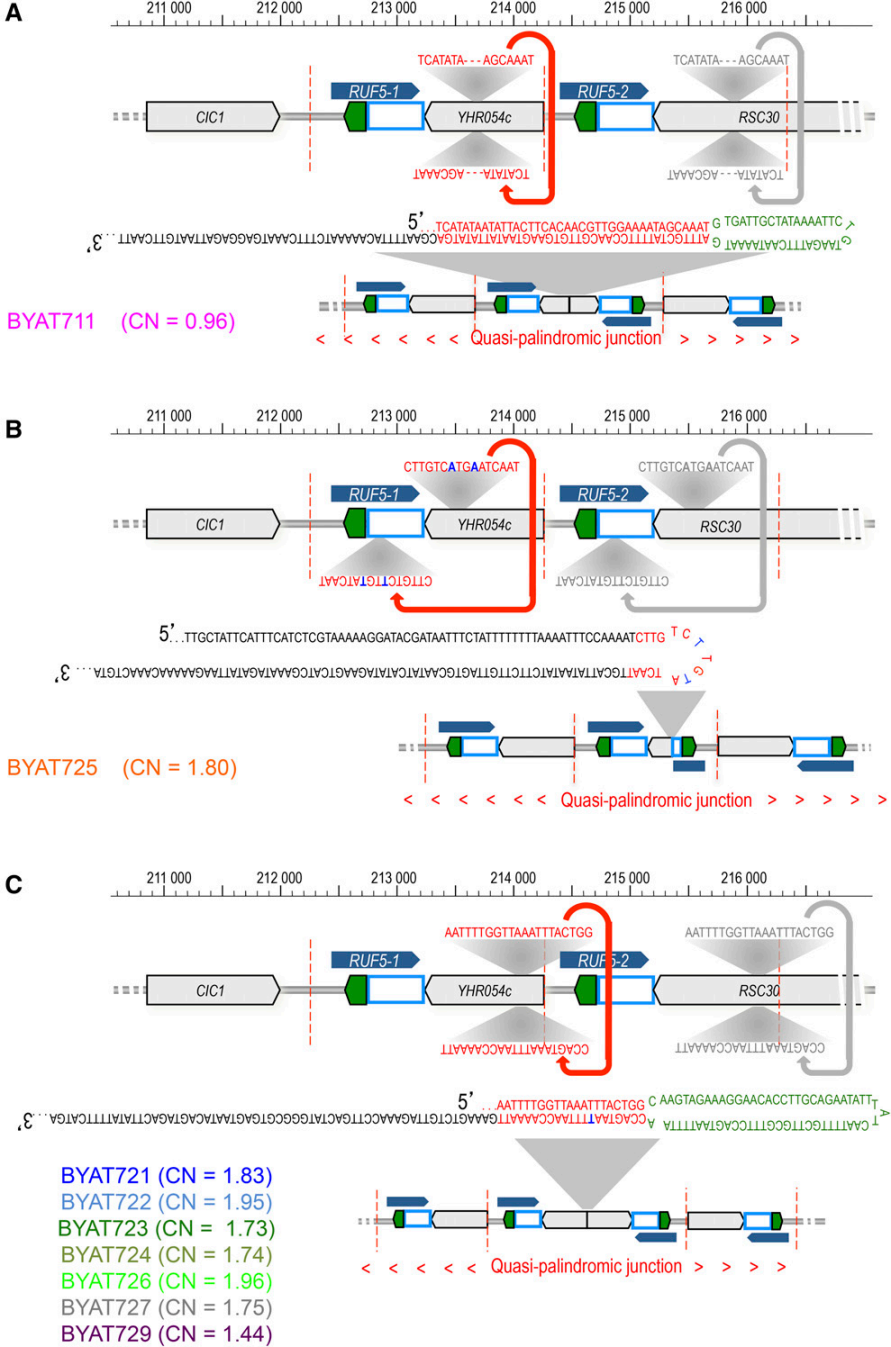
Novel junction sequences within the CUP amplicon. The map of the *CUP1* locus in the reference sequence is reproduced on top of each panel (same legend as Figure 3) with thick red arrows to locate the novel junctions discovered in each evolved mutant from whole-genome sequencing (reproduced in gray shade in the second repeat unit, for memory). For sequence reads across junctions, refer to Figure S3. The resulting palindromic structures formed are cartooned at the bottom of each panel (flanking units are in opposite orientation, red arrows). (A) The quasi-palindromic sequence identified in BYAT711 joined the two opposite DNA strands in *YHR054c*. Red letters: palindromic sequence, green letters: 38 nucleotides-long nonpalindromic interval. (B) The quasi-palindromic sequence identified in BYAT725 occurred between two 16-nucleotides-long, nearly identical inverted sequences in *YHR054c* and ARS810 (red letters, sequence differences between the two loci in blue). These two sequences are separated from each other by 745 nucleotides on the reference sequence. (C) The junction sequence identified in mutants BYAT-721, -722, -723, -724, -726, -727, and -729 joined the two opposite DNA strands at the beginning of *YHR054c*. Red letters: palindromic sequence, green letters: 63 nucleotides-long nonpalindromic interval. Note the single mismatch within the palindromic sequence (blue letters). CN, total copy number of the junction sequence in the cell as determined from sequence coverage.

All of the above data converge to indicate the presence of grossly altered chromosomal structures, the precise mapping of which is necessary to understand their origin. In a first step in this direction, we searched for novel junctions in the sequences of evolved mutants that could be indicative of chromosomal breakpoints. To do so, all unmapped reads of each mutant were assembled as described in the *Materials and Methods*, and new contigs with sufficient numbers of reads were compared to the reference sequence. To our surprise, we discovered quasi-palindromic sequences corresponding to the *CUP1* locus in all mutants. Each quasi-palindromic sequence was supported by a very large number of sequence reads (633–1494), leaving no possible doubt about their existence (Figure S3). Three distinct sequences were found in total, each specific to one or to a series of mutants (Figure 4). Two of them (Figure 4, A and C) correspond to the junction between the two opposite DNA strands within *RSC30* or more likely within one copy of *YHR054c*, leaving a single strand loop of 38 nt (mutant BYAT711) or 63 nt (all mutants of the BYAT721–729 series, except BYAT725). These junctions occurred without sequence homology and probably correspond to an inverted template switching during the replication of one or a few repeated copies of the *CUP1* locus. The third quasi-palindromic sequence, only observed in BYAT725, corresponds to the junction between two short (16 bp), nearly identical sequences in opposite orientation within the 2 kb repeat unit, the first one within *RSC30* (or *YHR054c*) and the second one upstream of the *CUP1* gene itself (Figure 4B). These two motifs are separated from each other by 745 nt on the reference sequence, leaving a nonpalindromic segment of equivalent size in the junction. We have verified that each quasi-palindromic sequence was not only specific to one type of mutant (not found in others) but also absent from genomes of other strains previously sequenced at high coverage (BYAT580-345, BYAT581-345, and BYAT583-345, Thierry *et al.* 2015).

Given the high numbers of CUP repeats in evolved mutants, it was particularly interesting to determine the copy number of each novel junction (deduced from sequence read coverages, see Figure S3). We found the BYAT711 junction present at normal haploid coverage (0.96 ×), indicating a single occurrence for the entire genome, a figure to be related to the presence of a single expanded *CUP1* locus in this genome (on the macrotene chromosome). In contrast, the two other junctions (in BYAT725 and the BYAT721–729 series) were present at nearly double coverage (see Figure 4), suggesting two occurrences per genome in these mutants, again correlated to the presence of two expanded *CUP1* loci (one in each macrotene chromosome). This correlation strongly suggests the presence of one quasi-palindromic junction within each expanded *CUP1* locus, the rest of the locus being made of direct tandem repeats of the 2 kb CUP amplicon, an important point for consideration when elucidating the origin of the novel chromosomal structures formed (see below).

In addition to the *CUP* junctions, another novel junction sequence was also discovered from complete genome sequencing of mutant BYAT725 (see Figure S4A). It joins a telomeric repeat (C_1-3_A)_n_ to nucleotide 123,222 within *YHR008c* (the left boundary of amplicon VIII-F). This sequence has a copy number of only 0.63 (as judged from sequence coverage), which is probably an underestimate given the difficulty of mapping sequence reads including short telomeric repeats. The fact that this junction could only be found in mutant BYAT725 is consistent with the presence of amplicon VIII-F in this mutant only, and offers independent confirmation of its existence (compared to VIII-E). Other junction sequences expected from the structures of macrotene chromosomes (see below) fall within δ elements, excluding their possible reassembly from shotgun sequence reads. One of them, joining *YHRW*δ*7* with *YHRC*δ*3* could, however, be directly verified by sequencing a PCR amplification product from mutant BYAT711 (Figure S4B).

### Topological structures and origin of macrotene chromosomes

**Figure 5:**
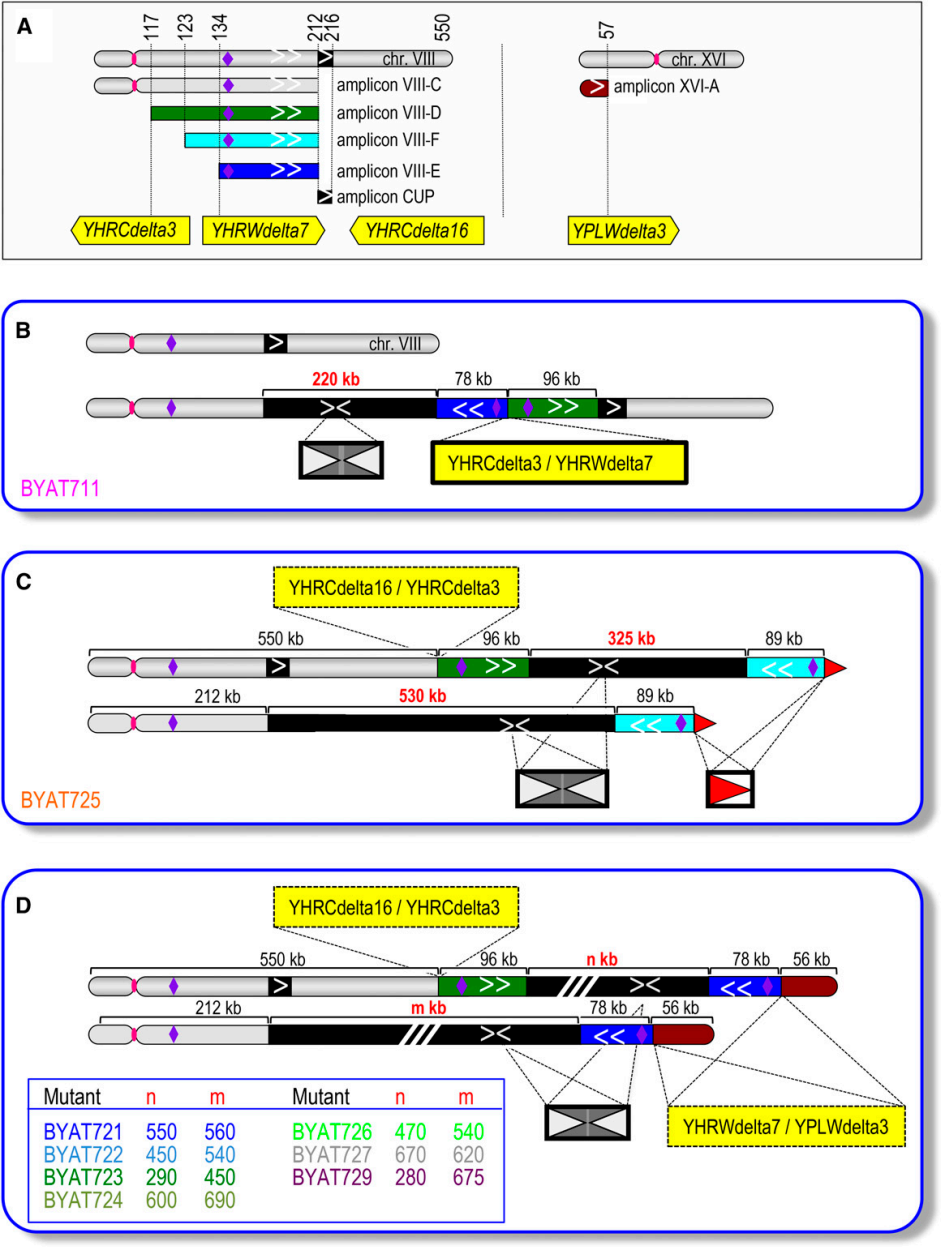
Deduced structures of macrotene chromosomes in evolved mutants. (A) Summary of amplicons with their sequence coordinates along chromosomes (in kb) and remarkable elements at their boundaries (yellow arrowhead boxes). Internal double arrows indicate orientation relative to the reference sequence. Pink tightening: centromere; black box: *CUP1* locus, purple diamond: *Y. lipolytica* Asn-RS gene. (B) Scheme of the normal and macrotene chromosomes in mutant BYAT711. Gray cylinders, original chromosome sequences; colored boxes, extra copies of amplicons. Novel junctions at breakpoints are indicated by rectangles (thick borders, experimentally determined sequences; dotted borders, hypothetical). Gray, quasi-palindromic junctions within the CUP amplicon (see Figure 4 and Figure S3); yellow, δ elements (see Figure S4). The macrotene chromosome drawn represents one of the four logical possibilities, depending upon the orientation of the novel VIIID-VIIIE segment and the relative positions of the normal and expanded *CUP* loci. (C) Scheme of the two macrotene chromosomes in mutant BYAT725. Same legend as B. Rectangle with red arrow: junction with telomeric repeats (see Figure S4). (D) Scheme of the two macrotene chromosomes in mutants BYAT721, -722, -723, -724, -726, -727, and -729. Same legend as B. All mutants share the same topological structures but differ from one another by the sizes of the two expanded *CUP* loci (insert, sizes in kb; see Table 2).

With the above data, we were able to reconstitute the structures of the macrotene chromosomes in the different evolved mutants (Figure 5). Mutant BYAT711, which contains a single macrotene chromosome in addition to a normal chromosome VIII, represents the simplest case. The double sequencing coverage over the entire chromosome VIII reference sequence (see Figure 2) indicates that the *macrotene* chromosome contains an entire copy of chromosome VIII, plus additional segments being made of extra copies of amplicons VIII-D and VIII-E (one of each), and of the 220 kb-long new *CUP1* locus. The back-to-back junction between amplicons VIII-D and VIII-E within their inverted δ boundaries (demonstrated by direct sequencing, see Figure S4B), and the quasi-palindromic junction within the expanded *CUP1* locus, argue in favor of an inverted region inside the macrotene chromosome, as illustrated by Figure 5B. Four equivalent structures of this type remain logically possible with presently available data, depending upon the relative location of the expanded *CUP1* locus *vs.* the original one (arbitrarily shown to the left on Figure 5B) and the relative orientation of amplicons VIII-D and VIII-E between the two *CUP1* loci (see *Discussion*).

Deduction of the structures of the other mutants was more complicated because each exhibited two macrotene chromosomes, one of which containing the entire chromosome VIII (except its 13 kb-long right subtelomeric segment, see Figure 2) and the other entirely missing all sequences at the right of the *CUP1* locus. All these mutants showed two expanded *CUP1* loci in addition to the normal one (Figure 3C) and two quasi-palindromic junctions within the CUP repeat unit (Figure 4, B and C), strongly suggesting the presence of one such junction in each expanded locus. In mutant BYAT725, the junction between the left boundary of amplicon VIII-F and a telomeric (C_1-3_A)_n_ sequence (demonstrated on Figure S4A), suggests that the two extra copies of amplicon VIII-F were joined to the expanded CUP1 loci, serving as neo-telomeres for the two macrotene chromosomes as illustrated in Figure 5C. On the shorter macrotene chromosome, this structure is linked to the left part of chromosome VIII before the normal *CUP1* locus. On the longer macrotene chromosome, the same structure is associated with the extra copy of amplicon VIII-D, itself replacing the deleted 13 kb-long right subtelomeric segment (see Figure 2). This last junction, involving the δ*3* and δ*16* elements, is only hypothetical; the two others are experimentally supported.

The structure of all other mutants could be deduced from similar principles (Figure 5D), only assuming a novel junction between the extra copies of amplicons VIII-E and XVI-A, both present in duplicates (see Figure 2). The presence of δ elements in appropriate orientation at the boundaries of these amplicons supports this hypothesis. In this structural model, the differences in size between the macrotene chromosomes of the different mutants (Table 1) are entirely accounted for by the differences in size of their expanded *CUP1* loci, hence the copy numbers of the CUP amplicon (Figure 5D and Table 2).

**Figure 6:**
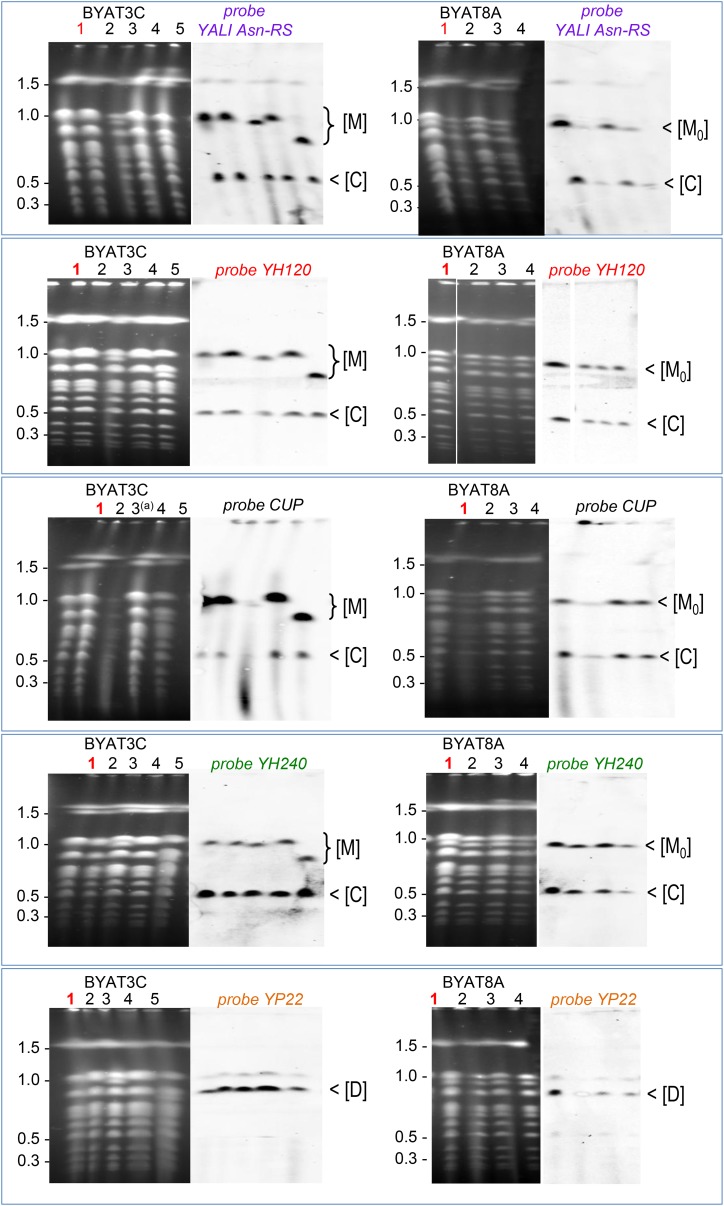
Pulsed-field gel electrophoresis (PFGE) of parental subclones. PFGE was run according to *Materials and Methods*. Left: ethidium bromide fluorescence, size scales in Mb calibrated from migration of natural *S. cerevisiae* chromosomes. Right: hybridization with indicated probes. Refer to Figure 1 and Table S3 for probe definition and to Figure S1B for origin of subclones of parental strains BYAT3C and BYAT8A. Subclones numbered “one” (bold red) were used for evolutionary experiments. (a), degraded DNA sample; [C], normal chromosome VIII; [D], normal chromosome XVI; [M], macrotene chromosomes; [M_0_], initial macrotene chromosomes (see Figure 8).

The formation of such complicated chromosomal structures remains, of course, difficult to imagine. In order to identify possible intermediate steps in their formation, we analyzed several subclones of each parental strain before the evolution experiments (see Figure S1B). The results proved to be highly interesting (Figure 6). All five subclones of *BYAT3C* and all four subclones of *BYAT8A* (including subclones numbered “one,” used to initiate the evolutionary experiments in both cases), revealed two bands hybridizing with the *YALI* Asn-RS probe, one corresponding in size to the normal chromosome VIII and the other to a macrotene chromosome. In other words, disomy and the formation of a first macrotene chromosome occurred very early in the haploid clones after meiosis. Both bands hybridized with the CUP probe as well as with the two probes on its left (YH120) and right (YH240) sides. None hybridized with the chromosome XVI probe. Subclone one of *BYAT3C* is indistinguishable from evolved mutant BYAT711, in agreement with the homogeneity of the eight evolved mutants of this experiment (see Figure 1A). The mutational event(s) at the origin of these evolved mutants must, therefore, have taken place very early during the cultivation of strain *BYAT3C*. Note, however, the different macrotene chromosomes in subclones three and five compared to subclones one, two, and four. The intense hybridization with the CUP probe suggests that massive amplification occurred in all of them (the DNA of subclone three was unfortunately partly degraded).

The four subclones of BYAT8A showed similar patterns (Figure 6), also suggesting that early mutational event(s) of a similar nature took place during cultivation of strain *BYAT8A*, generating a macrotene chromosome in addition to a normal size chromosome VIII (disomy). In this case, however, the *CUP1* locus was not amplified (as judged from hybridization intensity), an important indication of possible underlying mechanisms (see *Discussion*). The clear differences observed between the PFGE pattern of subclone one, and those of the evolved mutants derived from it (see Figure 1), demonstrates that the expansion of the *CUP1* locus and the replacement of the normal chromosome VIII copy by a second macrotene chromosome corresponded to subsequent mutational events, which occurred during this evolutionary experiment. The differences between BYAT725 and the other mutants indicate that at least two independent mutational events of similar type must have taken place during this evolutionary experiment.

### Stability of amplified structures and macrotene chromosomes

**Figure 7:**
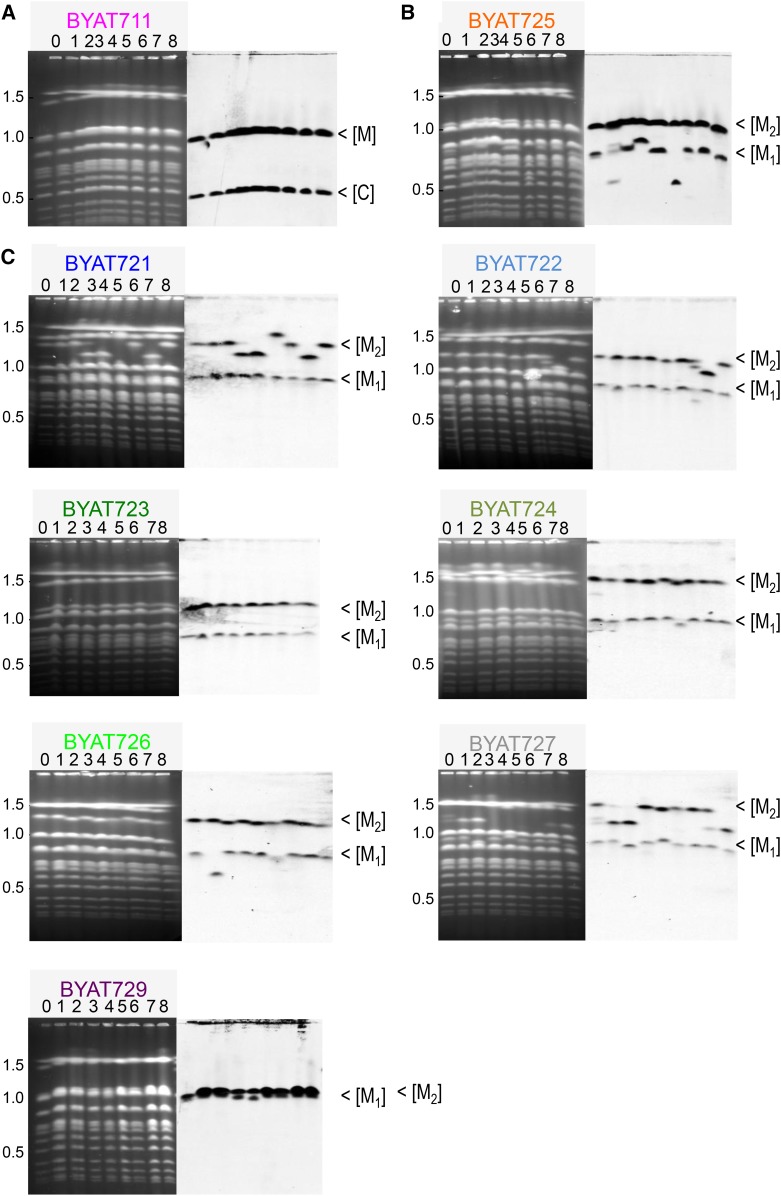
Stability of evolved mutants. Pulsed-field gel electrophoresis (see *Materials and Methods*) of original mutant (lane 0, compare to Figure 1) and its subclones after ∼25 additional generations on YPD medium (lanes 1–8). Left: ethidium bromide fluorescence, size scale in Mb calibrated from migration of natural *S. cerevisiae* chromosomes. Right: hybridization with probe YH120 (see Figure 1 and Table S3). (A) mutant from BYAT3C. (B and C) Mutants from BYAT8A. [C], normal chromosome VIII; [M], *macrotene* chromosome; [M_1_] initial size of shorter macrotene chromosome; [M_2_] initial size of longer macrotene chromosome.

To characterize this important point, we examined eight subclones from each evolved mutant (Figure 7). According to the protocol used (see Figure S1C), each subclone has underwent ∼25 additional generations on YPD medium from the original mutant. The result showed complete stability for BYAT711 within the limit of the experiment (eight subclones indistinguishable from one another and from the original mutant), whereas some instability was observed for the mutants derived from *BYAT8A*. In BYAT725, the large macrotene chromosome (M2) appeared more stable than the small one (two reductions, one expansion). The other mutants seemed to differ from one another but, in total, the large macrotene chromosomes appeared less stable (seven reductions and one expansion) than the small ones (two or three reductions, one expansion). Mutants BYAT723 and BYAY729 looked completely stable within the sampling limit tested. Overall, the different mutants appeared to be sufficiently stable to transmit their complex structures unchanged to the majority of their progeny (57/72) after ∼25 generations in rich medium. We did not determined the structures of the variant subclones, but it is possible that the differences in size correspond to instability within the new expanded *CUP1* loci. Most variants corresponded to shortening of the macrotene chromosomes, but two cases of size expansion were also visible (BYAT725 M1 and BYAT721 M2, see Figure 7).

## Discussion

The first example of a rearrangement altering gene order along a chromosome was discovered long ago in *Drosophila* (Sturtevant 1921), and the importance of this phenomenon and of gene copy number variations in the evolution of normal or altered genomes is now fully recognized (El-Mabrouk and Sankoff 2012). Thus, the extensively reshuffled chromosomal structures observed in the present study represent yet another example of these wider mechanisms, but have novel aspects worth considering for our general understanding. First, the very high genetic amplifications obtained (up to 880 copies of the 2 kb-long *CUP1* repeat unit) did not correspond to the phenotypic selection applied. The *CUP1* gene encodes a metallothionein (Winge *et al.* 1985). Among yeasts, it is uniquely found in *S. cerevisae* and related *Saccharomyces sensu stricto* species (Byrne and Wolfe 2005) and, thus, likely corresponds to a recent horizontal acquisition (from an as yet unidentified source) or *de novo* gene formation. Its increased copy number has been known to confer increased resistance to copper salt toxicity for a long time (Fogel and Welch 1982; Welch *et al.* 1983; Chang *et al.* 2013; Zhao *et al.* 2014); however, copper salts were not used in our experiments. The selection was based on the transgenic Asn-RS gene, encoding a tRNA synthetase and located 70 kb upstream of the *CUP1* locus. This gene was found to rise to a maximum of only five copies per cell; this was the result of large segmental duplications and chromosomal disomy, and not single gene amplification. Second, evolved mutants with restored fitness were obtained by the formation of grossly altered chromosomal structures, generally considered as phenotypically deleterious or prone to induce chromosome instability, such as aneuploidy, the presence of large inverted repeats, excessive chromosome length, or numerous tandem repeats. The effects of aneuploidy have been extensively examined in yeast (Torres *et al.* 2007). Frequently observed in mutants affected in the cytokinesis motor (Rancati *et al.* 2008), aneuploidy is generally compensated for by lower gene expression in natural yeast strains (Hose *et al.* 2015), and mutations in several genes or intergenic regions were previously reported to increase aneuploidy tolerance (Torres *et al.* 2010). We have verified the sequences of these genes and intergenes in all our evolved mutants, but could not find nucleotide substitutions or indels in any of them. We, therefore, consider that the chromosome VIII disomy observed is intrinsically linked to the mechanism of formation of the macrotene chromosomes, rather than being the result of phenotypic selection.

Compared to classical adaptation experiments in which gradual fitness increase results from sequence alteration or low level amplification directly affecting the few genes of interest (Burke 2012; Dettman *et al.* 2012; Barrick and Lenski 2013; de Visser *et al.* 2014), the phenotypic restoration obtained here from severely altered genotypes proceeded by catastrophic events operating at the chromosomal scale (we have verified the absence of point mutation in the transgenic Asn-RS locus in all evolved mutants studied). Contrary to chromothripsis (Zhang *et al.* 2013) observed in cancer cells, the altered chromosomal structures observed here do not correspond to massive fragmentation (no additional breakpoints beside those mentioned in *Results* were observed across the genomes of our evolved mutants), but can be interpreted by the interactions between a few anomalous events during replication and/or segregation of chromosomes, including the formation of quasi-palindromic junctions associated with the massive tandem expansion of flanking repeats. The resulting structures are complex macrotene chromosomes that are sufficiently stable to propagate unchanged over dozens of successive generations, despite the expected problems mentioned above.

**Figure 8:**
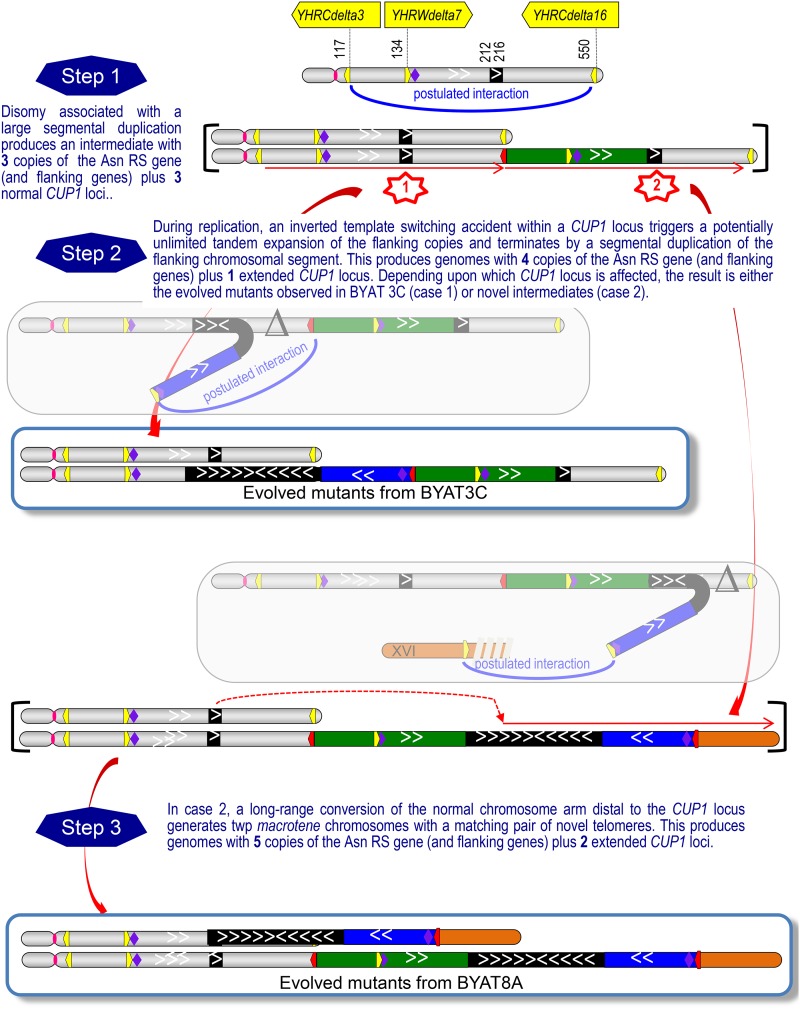
Model for the formation of macrotene chromosomes with massively amplified *CUP1* loci. Colored symbols of amplicons and chromosomal segments as in Figure 5. Step 1: The model postulates the formation of a first intermediate macrotene chromosome (equivalent to M_0_ in Figure 6) concomitant with disomy (shown between brackets). This macrotene chromosome corresponds to a long segmental duplication between two δ elements (top blue line), hence contains two normal *CUP1* loci (black rectangles) and two copies of the asparagine tRNA synthetase gene (purple diamonds). The chimeric δ formed at the junction is symbolized by the red arrowhead box. Step 2: A quasi-palindromic junction is accidentally generated (red stars) by a template switching event within one or the other normal *CUP1* locus, initiating a cascade of amplifications affecting the *CUP1* locus and its flanking segment (amplicon VIII-B) terminated by interactions between δ sequences (see *Discussion*). If the left *CUP1* locus is affected (case 1), the process may be terminated by the interaction between the two cooriented δ elements drawn, generating an internal deletion and placing amplicons VIII-D and VIII-E (green and blue rectangles, respectively) in the opposite orientation (this is the topology observed in mutant BYAT711, see Figure 5B). If the right *CUP1* locus is affected (case 2), the same mechanism results in the same δ element interacting with another structure to form a new telomere, hence deleting the distal part of the initial macrotene chromosome (demonstrated in Figure 2A). This interaction may involve amplicon XVI-A (as demonstrated in Figure 2B for mutants BYAT721, 722, 723, 724 726, 727, and 729, shown here) or the direct junction with a telomeric sequence (as demonstrated in Figure S4 for mutant BYAT725, not shown here). In both cases, a third step is required to transform the intermediate with one macrotene chromosome (under brackets) into the final structures observed in mutants having two macrotene chromosomes (see *Discussion* for mechanisms).

The process by which these complex structures form is very interesting. Although not directly demonstrated experimentally, a coherent model can be proposed after detailed examination of the different chromosomal topologies observed in the different evolved mutants and their parental strains (Figure 8). The model postulates the formation of an intermediate macrotene chromosome VIII, based on classical segmental duplication mechanisms (Koszul *et al.* 2004, 2006; Payen *et al.* 2008), nearly twice the size of the original chromosome VIII, in addition to the concomitant disomy of that chromosome. Such a structure was observed in the early subclones of the parental strain BYAT8A prior to starting the evolutionary experiment (see Figure 6), indicating a very early mutational event. How the disomy and large segmental duplication are mechanistically related remains unclear, but it should be noted that a similar association was previously observed at the origin of a macrotene chromosome IV in diploid cells (Thierry *et al.* 2015). In this intermediate macrotene chromosome VIII, the *CUP1* locus is not amplified (two normal loci coexist) and the Asn-RS gene is present in three copies per cell, a number likely sufficient to partially restore growth fitness (see Thierry *et al.* 2015 for discussion of phenotypes). The beauty of the model is that, at this stage, it postulates a unique molecular mechanism to explain the diversity of all of the final structures observed: the accidental formation of a quasi-palindromic junction within one or the other of the two normal *CUP1* loci. The absence of, or the limited, sequence homology at these junctions (see Figure 4) suggests accidental template switching events during replication. The presence of only one such junction in each amplified *CUP1* locus suggests that this event is the triggering step for the massive amplification of the flanking copies, which may simply result from a rolling-circle type of mechanism generated by the interaction between two replication bubbles, as previously suggested (Brewer *et al.* 2011; Thierry *et al.* 2015). The presence of a replication origin (ARS810) within each *CUP1* repeat (Nieduszynski *et al.* 2007), and the fact that the parental cells are severely stressed (Thierry *et al.* 2015), probably contribute to increase the frequency of such events. The rolling-circle amplification may eventually terminate in a segmental duplication of the chromosomal segment flanking the *CUP1* locus (itself limited by the possible interaction between δ elements as a means to regenerate a continuous chromosome, Figure 8). Depending on whether the initial template switch in a *CUP1* repeat occurs in the left or the right *CUP1* locus (which should have equivalent probability), two distinct topological situations follow that account for the mutants observed. In the first case, an internal deletion occurs, keeping the right chromosome end intact. The result is the structure observed in mutant BYAT711 (and likely in all other mutants of the BYAT3C evolutionary experiment, see Figure 1). In the second case, the equivalent deletion eliminates the right chromosome end, necessitating compensation via the addition of a new telomeric structure. The junction with the telomeric (C_1-3_A)_n_ repeats in BYAT725, or with a segmental duplication of the left subtelomeric region of chromosome XVI in other mutants, fulfills this need (see Figure 5).

The only question left with this model is why mutants corresponding to the second case (amplification of the right *CUP1* locus seen in the BYAT8A mutants) have two macrotene chromosomes, while mutant BYAT711, which corresponds to the first case, keeps a normal chromosome. The presence of five copies of the Asn-RS gene as opposed to four may provide some phenotypic advantage but the effect is probably minor given our previous data (Thierry *et al.* 2015). In addition, this hypothesis does not explain why mutant BYAT711 is stable. We prefer to think that the basic reason for replacing the normal chromosomal copy with a second macrotene chromosome (with identical topology to the first) is the difficulty of maintaining two chromosome homologs with distinct telomeres. To our knowledge, this idea has not previously been proposed before but may warrant further study. The correction of the normal chromosome to a copy of the macrotene chromosome (with duplication of the amplified *CUP1* locus) is not difficult to imagine if one thinks about the long-range loss-of-heterozygosity phenomena observed in chromosomes of yeast hybrids (Leh-Louis *et al.* 2012; Morales and Dujon 2012). The opposite correction is obviously possible, but it would result in a cell with two normal chromosome copies expected to have a severely deleterious phenotype (only two Asn-RS genes).

The gene amplifications observed here largely surpass previously known figures of tandem gene arrays in number and, as such, may be regarded as experimental curiosities. However, recent unpublished data of the 1002 yeast genomes sequencing project (http://1002genomes.u-strasbg.fr) reveal the existence of a number of natural *S. cerevisiae* isolates exhibiting amplified *CUP1* loci, with total copy numbers approaching those of our mutants, suggesting that the same mechanism may operate in nature (J. Schacherer, personal communication). The fact that such events occur in the absence of direct phenotypic selection (the massive amplification of the *CUP1* gene did not increase the tolerance of our mutants to copper salts compared to parental strains with 16 copies, data not shown), suggests that they do not generate an important fitness cost, a phenomenon that has already been mentioned for gene amplification in plants (Vila-Aiub *et al.* 2014), and which is further documented here by the important stability of our mutants.

## Supplementary Material

Supplemental Material
