# Supplementary material for: Massive Amplification at an Unselected Locus Accompanies Complex Chromosomal Rearrangements in Yeast
Source: G3 (Bethesda). 2016 Mar 4;6(5):1201–15. doi: 10.1534/g3.115.024547 (PMC4856073; doi:10.1534/g3.115.024547)
Supplement: Supplemental Material [file supp_6_5_1201__index.html]

Massive Amplification at an Unselected Locus Accompanies Complex Chromosomal Rearrangements in Yeast — Supplemental Material 

# Massive Amplification at an Unselected Locus Accompanies Complex Chromosomal Rearrangements in Yeast

## Supplemental Material for Thierry, Khanna, and Dujon, 2016

**Files in this Data Supplement:**

- File S1 - Construction of parental strains and isolation of evolved mutants. (.pdf, 73 KB)
- Figure S1 - Scheme of experiments. (.pdf, 1,393 KB)
- Figure S2 - Copy number variation along chromosomes in evolved mutants. (.pdf, 492 KB)
- Figure S3 - Sequence reads covering the novel quasi-palindromic junctions within the CUP amplicon. (.pdf, 1,075 KB)
- Figure S4 - Amplicon boundaries and novel junctions sequenced. (.pdf, 142 KB)
- Figure S5 - Sequence analysis pipeline. (.pdf, 87 KB)
- Table S1 - Origin and genotypes of strains. (.pdf, 110 KB)
- Table S2 - Molecular analysis of meiotic products from BYAT580-0 and BYAT580-200. (.pdf, 114 KB)
- Table S3 - Synthetic oligonucleotide primers used in this work. (.pdf, 89 KB)
