# Supplementary material for: Massive Amplification at an Unselected Locus Accompanies Complex Chromosomal Rearrangements in Yeast
Source: G3 (Bethesda). 2016 Mar 4;6(5):1201–15. doi: 10.1534/g3.115.024547 (PMC4856073; doi:10.1534/g3.115.024547)
Supplement: Supplemental Material [file supp_g3.115.024547_FileS1.pdf]

File S1: Construction of parental strains and isolation of evolved mutants

Transgenic *S. cerevisiae* strains in which the essential Asn RS gene (*DED1*) has been replaced by its ortholog *(YALI0E05005g)* from *Y. lipolytica* showed a very reduced growth rate, making them delicate to manipulate because spontaneous mutants with restored fitness tend to rapidly accumulate in cultures. In order to obtain haploid strains bearing this genetic construct in their original form to start our experiments, we sporulated evolved diploids obtained from previous experiments (Thierry *et al.,* 2015), and isolated haploid segregants that were immediately frozen at -80°C after a minimal number of generations, as schematized by Figure S1. Parental strains and mutants are listed in Table S1. Genomic digests and hybridizations (shown by Table S2) were used to verify the molecular structure of each meiotic segregant before starting the experiments.

The diploid strain BYAT580-0 bears two normal chromosome VIII plus three copies of a large circular episome (amplicon VIII-A, Thierry *et al.,* 2015). Tetrads showed moderate size variation between ascospore colonies, consistent with a random segregation of the episome (Figure S1a). The haploid segregant BYAT580-0-3C proved devoid of episome (Table S2) and was selected for present experiments under the abbreviated name BYAT3C.

The diploid strain BYAT580-200 bears a *macrotene* chromosome VIII with 12 extra copies of amplicon VIII-B in addition to a normal chromosome VIII (Thierry *et al.,* 2015). Tetrads showed segregation between rapidly growing colonies, expected to carry the *macrotene* chromosome, and very slowly growing colonies expected to only carry the normal size chromosome (Figure S1a). This was directly verified for the haploid segregants BYAT580-200-8A and BYAT580-200-9B (Table S2). The first was selected for present experiments under the abbreviated name BYAT8A.

Frozen stocks of BYAT3C and BYAT8A were used to initiate experiments as illustrated by Figure S1b. After subcloning, five subclones of each strain were conserved, one of them serving to inoculate the evolutionary experiments by serial transfers on YPD as described in Methods. At each step, cultures were diluted and plated on YPD medium. Size heterogeneity of colonies and comparison with the control strain BYAT521 incubated in the same conditions suggested the appearance of faster growing mutants. For BYAT8A, a limited number of large colonies appeared among a large number of very small colonies, as expected of an evolving population. For BYAT3C, a much more homogeneous colony size was observed, suggesting that an early mutational event propagated in the population (see text for details). Eight large colonies, considered as evolved mutants, were picked up from each strain, immediately frozen at -80°C to prevent possible uncontrolled subsequent evolution and submitted to molecular analyses.
