# Supplementary material for: Massive Amplification at an Unselected Locus Accompanies Complex Chromosomal Rearrangements in Yeast
Source: G3 (Bethesda). 2016 Mar 4;6(5):1201–15. doi: 10.1534/g3.115.024547 (PMC4856073; doi:10.1534/g3.115.024547)
Supplement: Supplemental Material [file supp_g3.115.024547_FigureS1.pdf]

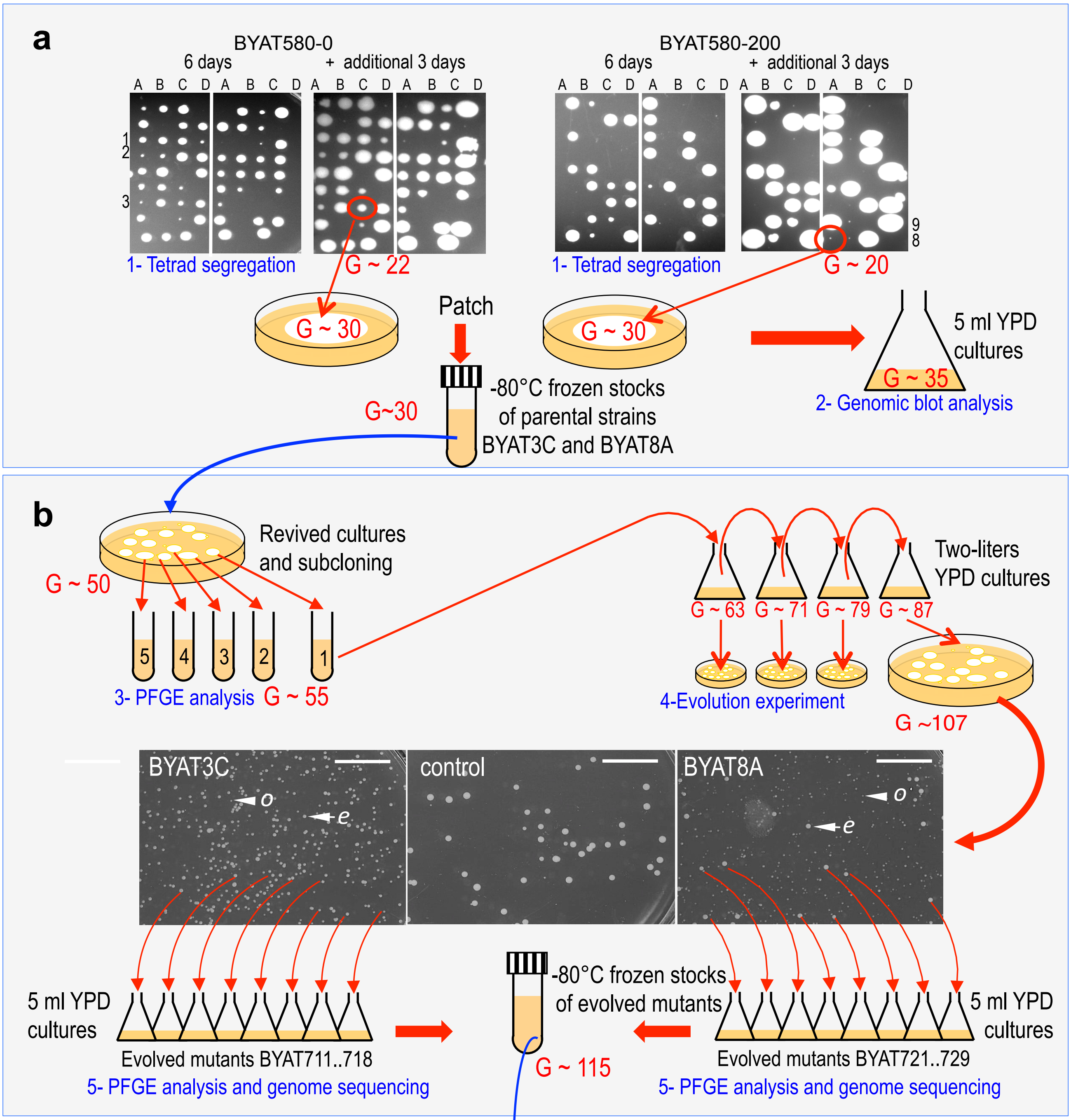

Figure S1: Scheme of experiments
(a) Construction and molecular verification of parental haploid strains (see Methods and text). Haploid segregants BYAT580-0-3C and BYAT580-200-8A (circled) were chosen for subsequent experiments (and renamed BYAT3C and BYAT8A, respectively). The approximate number of total generations from the original ascospore is indicated by **G**. Step 2 *"Genomic blot analysis"* refers to Table S2.

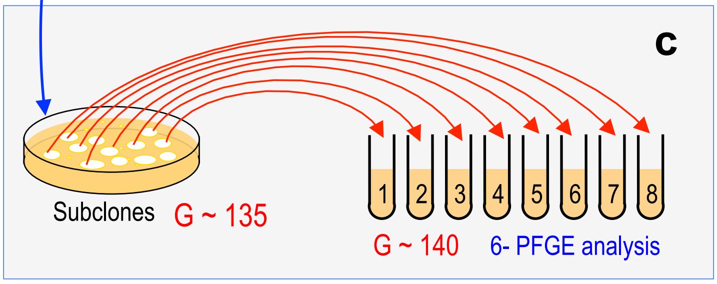

(b) Evolutionary experiments and analysis of evolved mutants. Subclones #1 of each strain were used to inoculate serial transfer cultures. Step 3 refers to Figure 6. Pictures of Petri dishes exemplify appearance of evolved mutants (*o:* original colony size, *e:* evolved mutant, scale bar: 1 cm). Control is BYAT521**.** Step 5 refers to Figures 1, 2, 3 and text. **(c)** Stability of evolved mutants. 8 subclones were picked up at random from each evolved mutants and grown for a few generations. Step 6 refers to Figure 7.
