# Supplementary material for: Massive Amplification at an Unselected Locus Accompanies Complex Chromosomal Rearrangements in Yeast
Source: G3 (Bethesda). 2016 Mar 4;6(5):1201–15. doi: 10.1534/g3.115.024547 (PMC4856073; doi:10.1534/g3.115.024547)
Supplement: Supplemental Material [file supp_g3.115.024547_FigureS2.pdf]

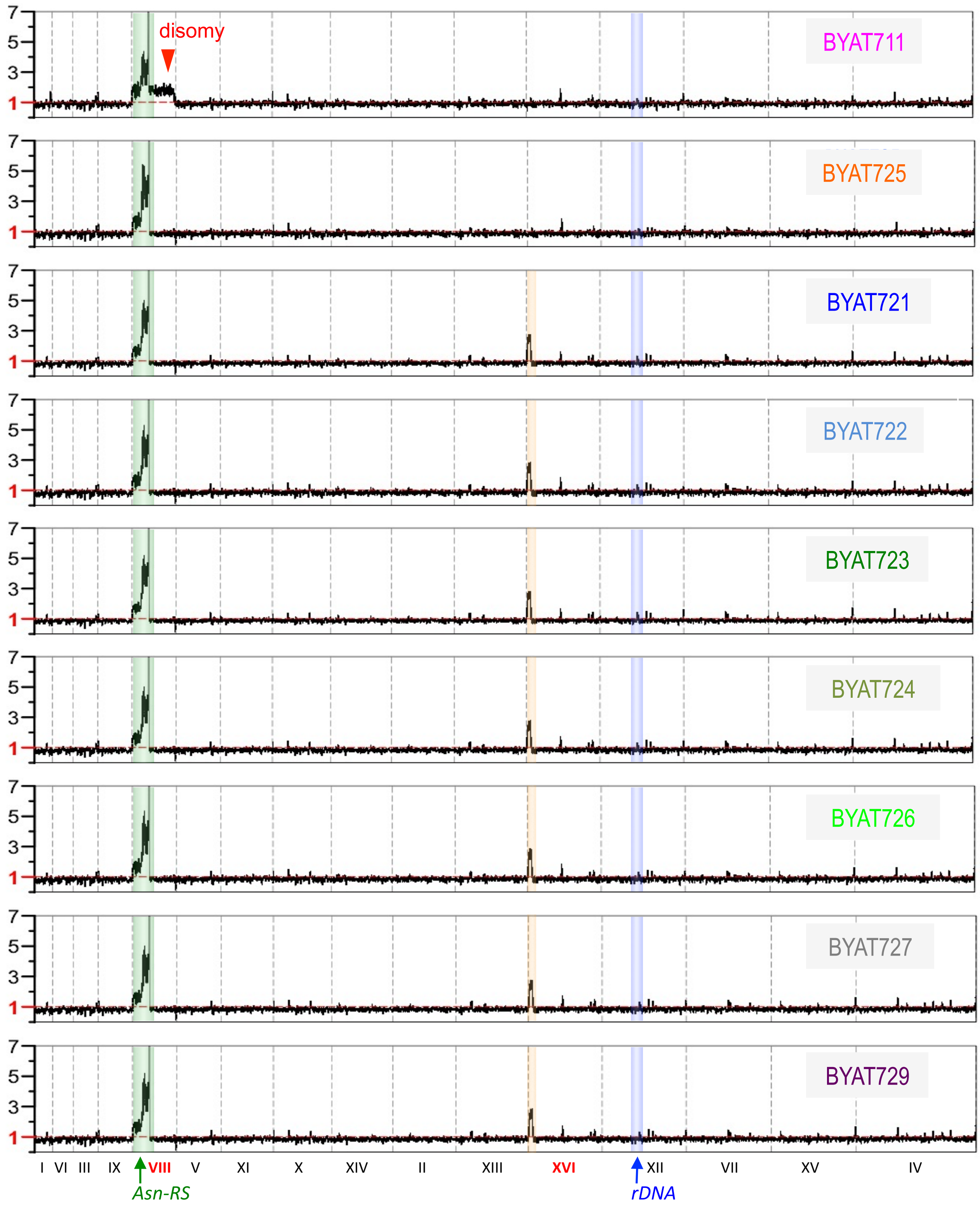

Figure S2 part A

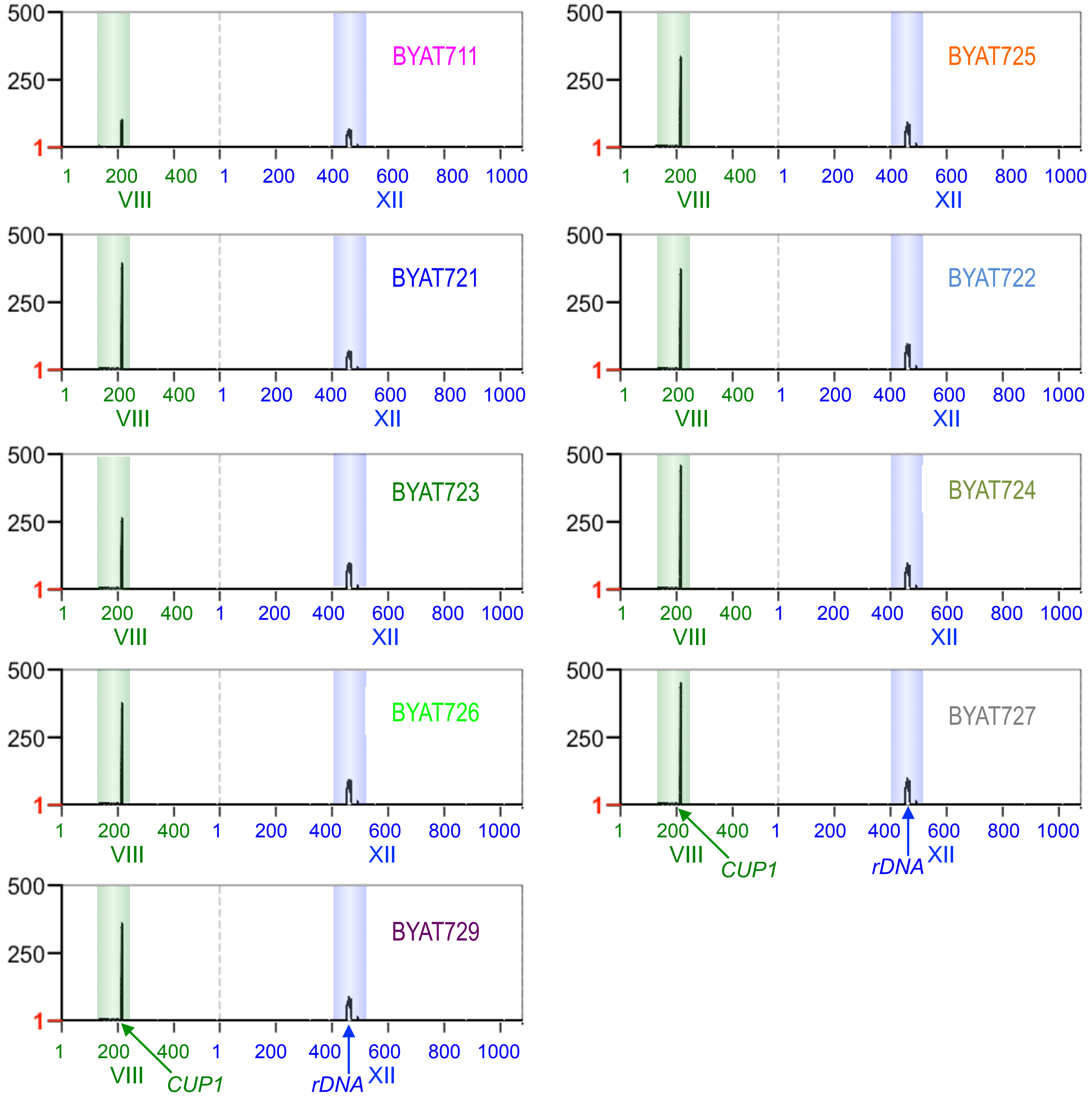

Figure S2 part B

Figure S2: Copy number variation along chromosomes in evolved mutants..

A: Low-order amplications along all chromosomes: For each sequenced strain, the copy number of each locus (ordinate) was plotted along the 16 chromosomes of the S288C reference sequence, ranked by increasing sizes (abscissa, roman numerals). Plots darwn using R version 3.1.2 (http://www.R-project.org/. Copy numbers were computed from local sequence coverage relative to the median coverage for that strain, and normalized to 1 (haploids) as indicated in Methods. Curves were generated using R version 3.1.2 (http://www.R-project.org). Curve smoothing (5,000 bp sliding-windows). Ordinates from 0 to 7. Amplifications of the rDNA locus (purple backgound) and *CUP1* locus (within green background) were artificially cut for drawing clarity. Refer to part B for actual figures. Position of the *YALI* Asn-RS gene is indicated (chromosome VIII). Segmental amplifications of interest in chromosomes VIII or XVI are highlighted by green or orange backgrounds, respectively.

B: High order amplifications along chromosomes VIII and XII: For each sequenced strain, the copy number of each locus (ordinate) was plotted along chromosomes VIII (left) and XII (right). Sequence coordinates in kb. Same legend as part A. Curve smoothing (1,500 bp sliding-windows). Ordinates from 0 to 500 in order to visualize amplifications of the *CUP1* locus (green background) and rDNA locus (purple background). The low-order segmental amplifications described in part A are not visible at this scale.
