# Supplementary material for: Massive Amplification at an Unselected Locus Accompanies Complex Chromosomal Rearrangements in Yeast
Source: G3 (Bethesda). 2016 Mar 4;6(5):1201–15. doi: 10.1534/g3.115.024547 (PMC4856073; doi:10.1534/g3.115.024547)
Supplement: Supplemental Material [file supp_g3.115.024547_FigureS3.pdf]

a

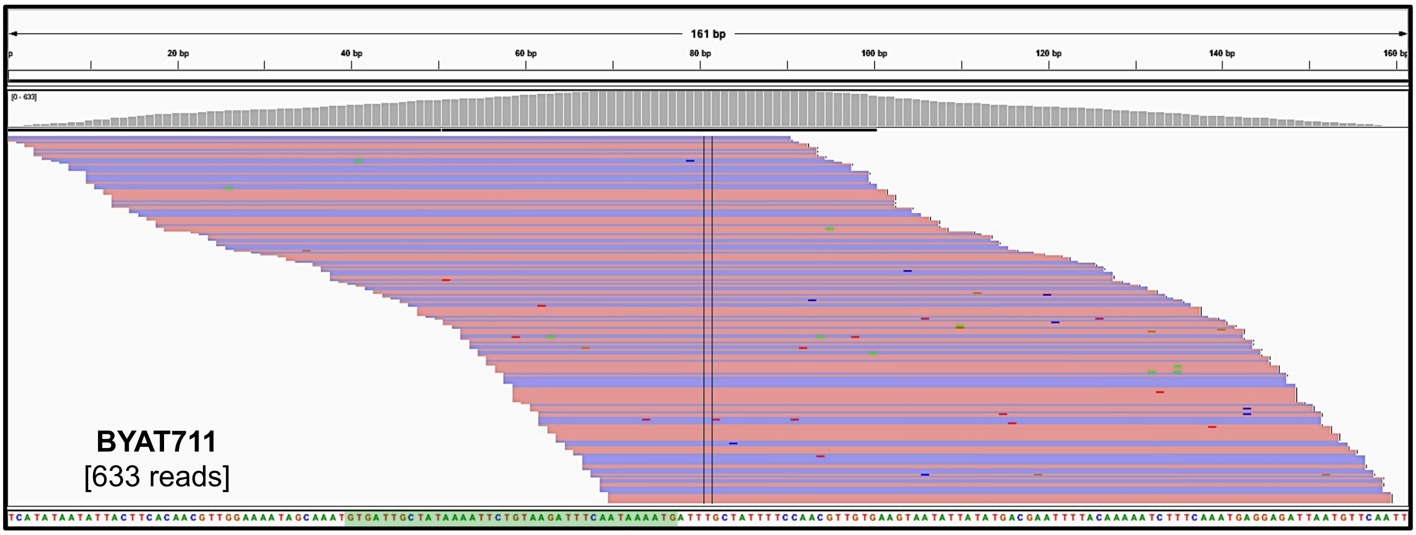
161 bp
20 bp
40 bp
60 bp
80 bp
100 bp
120 bp
140 bp
BYAT711
[633 reads]

b

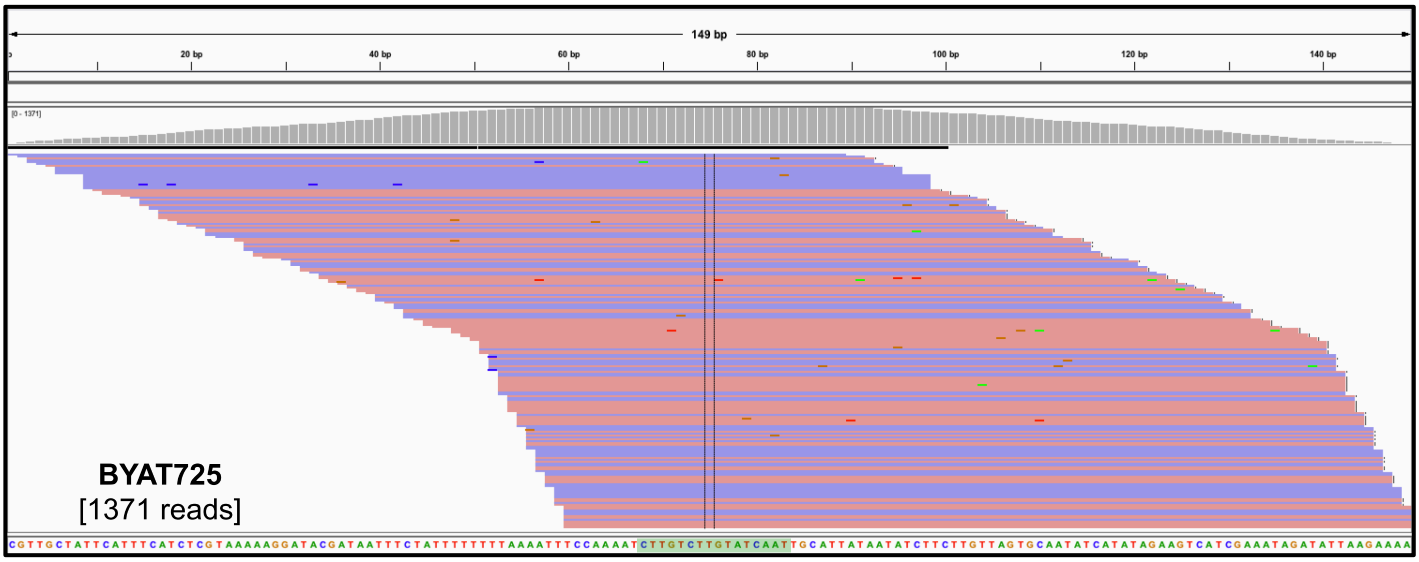
149 bp
20 bp
40 bp
60 bp
80 bp
100 bp
120 bp
140 bp
BYAT725
[1371 reads]

c

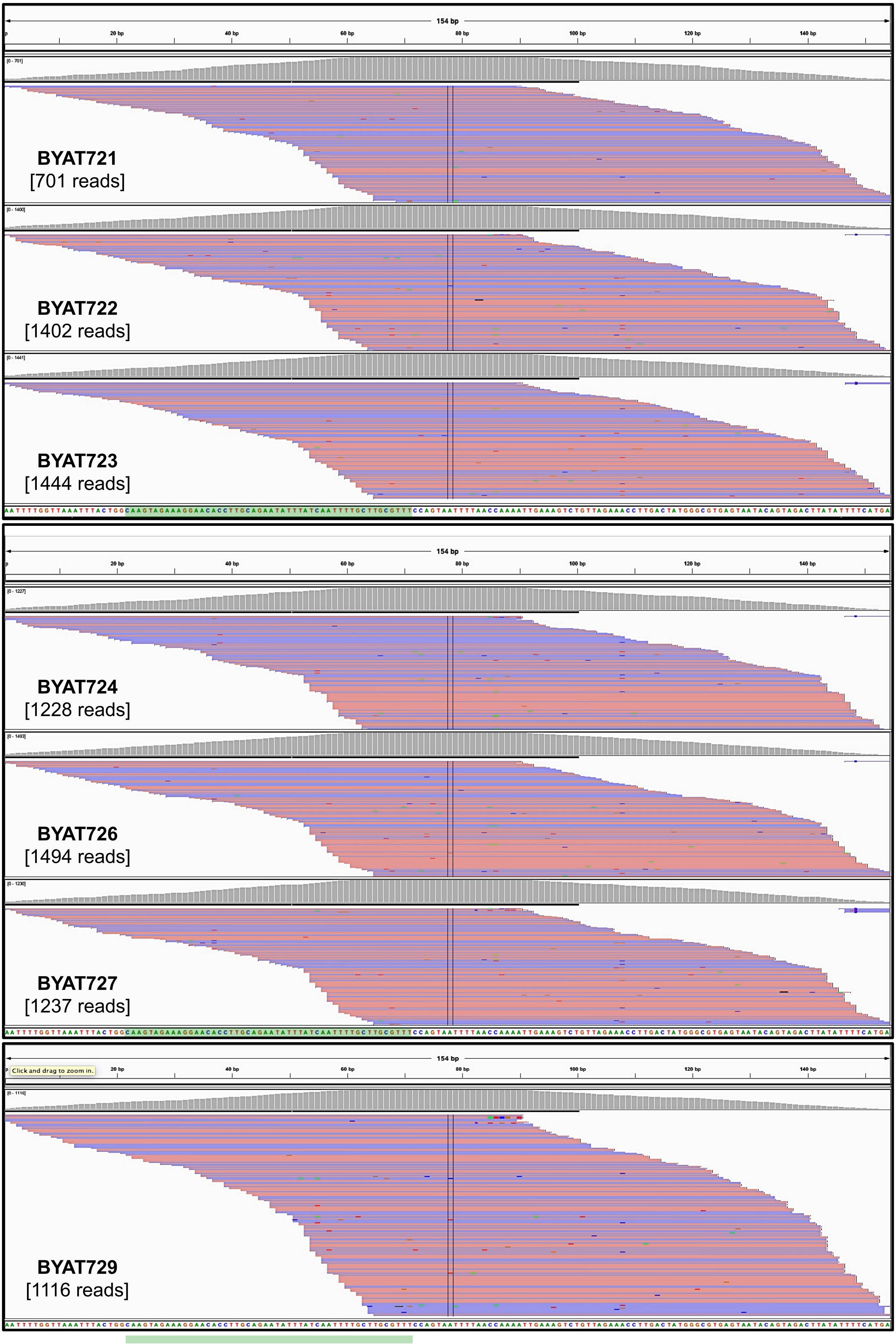
154 bp
20 bp
40 bp
60 bp
80 bp
100 bp
120 bp
140 bp
BYAT721
[701 reads]
BYAT722
[1402 reads]
BYAT723
[1444 reads]
BYAT724
[1228 reads]
BYAT726
[1494 reads]
BYAT727
[1237 reads]
Click and drag to zoom in.
BYAT729
[1116 reads]

Figure S3: Sequence reads covering the novel quasi-palindromic junctions within the CUP amplicon. For each evolved mutant, sequence reads were mapped using IGV (see Methods) against the corresponding junction sequence identified in Figure 4, serving as reference (**a:** BYAT711, **b:** BYAT725, **c:** BYAT721, -722, -723, -724, -726, -727 and -729). Total numbers of matching reads are indicated under brackets. Red: forward reads: blue: reverse reads. Total coverage at each position is shown on top (grey pyramids). The reference junction sequence is shown at bottom (see Figure 4). For a and c, green backgrounds highlight the central non-palindromic segments, flanking sequences are palindromic. For b, green background highlights the short inverted motif in the *CUP1* repeat unit, the left flanking sequence is included within the long non-palindromic interval between the two motifs, the right flanking sequence corresponds to the reverse complement of the reference sequence to the left of the first motif (see Figure 4).
