# Supplementary material for: Massive Amplification at an Unselected Locus Accompanies Complex Chromosomal Rearrangements in Yeast
Source: G3 (Bethesda). 2016 Mar 4;6(5):1201–15. doi: 10.1534/g3.115.024547 (PMC4856073; doi:10.1534/g3.115.024547)
Supplement: Supplemental Material [file supp_g3.115.024547_FigureS5.pdf]

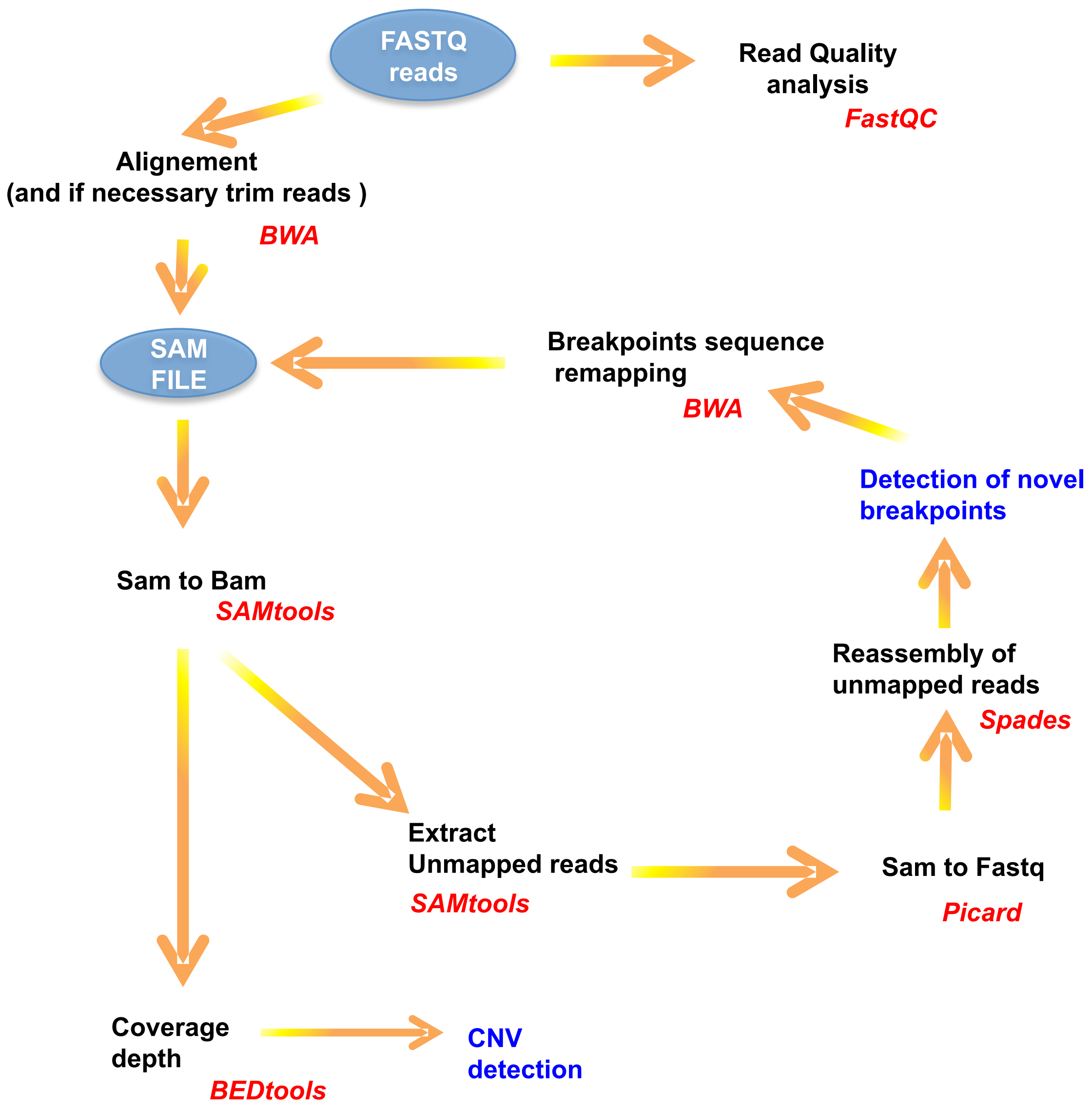


**Figure S5: Sequence analysis pipeline.**
Files are represented by blue ovals. Softwares and tools used at different steps are indicated in red, next to main action. See Methods for details.
