# Supplementary material for: Massive Amplification at an Unselected Locus Accompanies Complex Chromosomal Rearrangements in Yeast
Source: G3 (Bethesda). 2016 Mar 4;6(5):1201–15. doi: 10.1534/g3.115.024547 (PMC4856073; doi:10.1534/g3.115.024547)
Supplement: Supplemental Material [file supp_g3.115.024547_TableS1.pdf]

Table S1: Origin and genotypes of strains

Parental strains	Ploidy	Nuclear genotype	Origin
BYAT521	2n	*MATa/MATα ura3Δ0 /ura3Δ0 leu2Δ0/leu2Δ0 his3Δ1/his3Δ1 lys2Δ0/+ met15Δ0/+ YHR019c::YALI0E05005g-KanMX / +*	Thierry *et al.*, 2015
BYAT580-0	2n	*MATa/MATα ura3Δ0 /ura3Δ0 leu2Δ0/leu2Δ0 his3Δ1/his3Δ1 lys2Δ0/+ met15Δ0/+ YHR019c::YALI0E05005g-KanMX / YHR019c::YALI0E05005g-KanMX* (pAT260) [a]	Thierry *et al.*, 2015
BYAT580-0-3C *BYAT3C* [b]	n	*MATa ura3Δ0 leu2Δ0 his3Δ1 met15Δ0 YHR019c::YALI0E05005g-KanMX*	Meiosis of BYAT580-0
BYAT580-200	2n	*MATa/MATα ura3Δ0 /ura3Δ0 leu2Δ0/leu2Δ0 his3Δ1/his3Δ1 lys2Δ0/+ met15Δ0/+ YHR019c::YALI0E05005g-KanMX / YHR019c::YALI0E05005g-KanMX*	Thierry *et al.*, 2015
BYAT580-200-8A *BYAT8A* [b]	n	*MATa ura3Δ0 leu2Δ0 his3Δ1 YHR019c::YALI0E05005g-KanMX*	Meiosis of BYAT580-200

Evolved mutants	Ploidy	Nuclear genotype	Origin
**BYAT711**	n	*MATa ura3Δ0 leu2Δ0 his3Δ1 met15Δ0 YHR019c::YALI0E05005g-KanMX*	Evolved mutant of *BYAT3C*
BYAT712	n	*MATa ura3Δ0 leu2Δ0 his3Δ1 met15Δ0 YHR019c::YALI0E05005g-KanMX*	Evolved mutant of *BYAT3C*
BYAT713	n	*MATa ura3Δ0 leu2Δ0 his3Δ1 met15Δ0 YHR019c::YALI0E05005g-KanMX*	Evolved mutant of *BYAT3C*
BYAT714	n	*MATa ura3Δ0 leu2Δ0 his3Δ1 met15Δ0 YHR019c::YALI0E05005g-KanMX*	Evolved mutant of *BYAT3C*
BYAT715	n	*MATa ura3Δ0 leu2Δ0 his3Δ1 met15Δ0 YHR019c::YALI0E05005g-KanMX*	Evolved mutant of *BYAT3C*
BYAT716	n	*MATa ura3Δ0 leu2Δ0 his3Δ1 met15Δ0 YHR019c::YALI0E05005g-KanMX*	Evolved mutant of *BYAT3C*
BYAT717	n	*MATa ura3Δ0 leu2Δ0 his3Δ1 met15Δ0 YHR019c::YALI0E05005g-KanMX*	Evolved mutant of *BYAT3C*
BYAT718	n	*MATa ura3Δ0 leu2Δ0 his3Δ1 met15Δ0 YHR019c::YALI0E05005g-KanMX*	Evolved mutant of *BYAT3C*
**BYAT721**	n	*MATa ura3Δ0 leu2Δ0 his3Δ1 YHR019c::YALI0E05005g-KanMX*	Evolved mutant of *BYAT8A*
**BYAT722**	n	*MATa ura3Δ0 leu2Δ0 his3Δ1 YHR019c::YALI0E05005g-KanMX*	Evolved mutant of *BYAT8A*
**BYAT723**	n	*MATa ura3Δ0 leu2Δ0 his3Δ1 YHR019c::YALI0E05005g-KanMX*	Evolved mutant of *BYAT8A*
**BYAT724**	n	*MATa ura3Δ0 leu2Δ0 his3Δ1 YHR019c::YALI0E05005g-KanMX*	Evolved mutant of *BYAT8A*
**BYAT725**	n	*MATa ura3Δ0 leu2Δ0 his3Δ1 YHR019c::YALI0E05005g-KanMX*	Evolved mutant of *BYAT8A*
**BYAT726**	n	*MATa ura3Δ0 leu2Δ0 his3Δ1 YHR019c::YALI0E05005g-KanMX*	Evolved mutant of *BYAT8A*
**BYAT727**	n	*MATa ura3Δ0 leu2Δ0 his3Δ1 YHR019c::YALI0E05005g-KanMX*	Evolved mutant of *BYAT8A*
**BYAT729**	n	*MATa ura3Δ0 leu2Δ0 his3Δ1 YHR019c::YALI0E05005g-KanMX*	Evolved mutant of *BYAT8A*

All *S. cerevisiae* strains are S288c derivatives. **Bold underlined:** sequenced strains. (a) pAT260 is a replicative plasmid used for the construction of the original strain (see Thierry *et al.* 2015). (b) abbreviated names of, respectively, BYAT580-0-3C and BYAT580-200-8A, used throughout this article.
