# Supplementary material for: Massive Amplification at an Unselected Locus Accompanies Complex Chromosomal Rearrangements in Yeast
Source: G3 (Bethesda). 2016 Mar 4;6(5):1201–15. doi: 10.1534/g3.115.024547 (PMC4856073; doi:10.1534/g3.115.024547)
Supplement: Supplemental Material [file supp_g3.115.024547_TableS2.pdf]

Table S2: Molecular analysis of meiotic products from BYAT580-0 and BYAT580-200.

Strains	*Nsi* I digest probe *YALI* Asn-RS	*Bam* HI digest probe YHR020w	Conclusion
BYAT580-0	7.4 + 12.9	4.8 + 8.8	original construct + episome VIII-A
BYAT580-0-2A		4.8 + 8.8	original construct + episome VIII-A
BYAT580-0-2B		4.8 + 8.8	original construct + episome VIII-A
BYAT580-0-2C		4.8 + 8.8	original construct + episome VIII-A
BYAT580-0-2D		4.8 + 8.8	original construct + episome VIII-A
BYAT580-0-3A	7.4 + 12.9	4.8 + 8.8	original construct + episome VIII-A
BYAT580-0-3B	7.4 + 12.9	4.8 + 8.8	original construct + episome VIII-A
**BYAT580-0-3C**	**7.4**	**4.8**	**original construct**
BYAT580-0-3D	7.4 + 12.9	4.8 + 8.8	original construct + episome VIII-A
BYAT580-200	7.4 + 7.9	4.8 + 10.4	original construct + macrotene VIII-B
**BYAT580-200-8A**		**4.8**	**original construct**
BYAT580-200-8C		4.8 + 10.4	original construct + macrotene VIII-B
BYAT580-200-9A		4.8 + 10.4	original construct + macrotene VIII-B
BYAT580-200-9B		4.8	original construct
BYAT580-200-9D		4.8 + 10.4	original construct + macrotene VIII-B

Total DNA from diploid strains BYAT580-0 and BYAT580-200, and their haploid meiotic segregants were purified and analyzed by genomic blots. DNA were digested with either *Nsi*I or *Bam*HI as indicated, electrophorized and hybridized with gel-purified PCR probes corresponding to either the *Y. lipolytica YALI0E05005g* gene (*YALI* Asn-RS) or the *S. cerevisiae YHR020w* gene. The table indicates the size (in kb) of hybridizing fragments. For *Nsi*I digests hybridized with the *YALI* Asn-RS probe, the 7.4 kb band corresponds to the original non-amplified structure, the 12.9 kb band corresponds to the circular episome of amplicon VIII-A and the 7.9 kb band corresponds to the *macrotene* chromosome. For *Bam*HI digests hybridized with the *YHR020w* probe, the 4.8 kb band corresponds to the original non-amplified structure, the 8.8 kb band corresponds to the circular episome of amplicon VIII-A and the 10.4 kb band corresponds to the *macrotene* chromosome (see Supplementary Figure 7 of Thierry *et al.*, 2015 for details). Strains BYAT580-0-3C and BYAT580-200-8A were used as parental strains for the present work (respectively abbreviated as BYAT 3C and BYAT8A).
