# Supplementary material for: Massive Amplification at an Unselected Locus Accompanies Complex Chromosomal Rearrangements in Yeast
Source: G3 (Bethesda). 2016 Mar 4;6(5):1201–15. doi: 10.1534/g3.115.024547 (PMC4856073; doi:10.1534/g3.115.024547)
Supplement: Supplemental Material [file supp_g3.115.024547_TableS3.pdf]

Table S3 : Synthetic oligonucleotide primers used in this work.

primer n°	DNA sequence (5' - 3')	Chromo -some	5' coordinate and orientation	application
AT 261	CCGTAGATGGCAACAGATGA	VIII		*YALI* Asn-RS probe
AT 357	ATGAGTCAAAGTTCGTCTGA	VIII		*YALI* Asn-RS probe
AT 495	TCGGTCTACCAAAGACAG	VIII	239 125 -->	YH240 probe
AT 496	TAACAGGTGCTATACACA	VIII	240 106 <--	YH240 probe
AT 499	CTGCGGTAACAAGTCTG	VIII	212 772 (a) <--	CUP probe
AT 504	CATTCACCGGTACTTCCA	VIII	211 101 (a) -->	CUP probe
AT 512	CACGTGAATGTACAACTTC	VIII	120 027 -->	YH120 probe
AT 513	ACTTACCATTGGAACACTA	VIII	120 927 <--	YH120 probe
AT 514	GTAACCTAGCTGAATAAG	XVI	22 027 -->	YP22 probe
AT 515	AGTTAGGTTTTGTAGACTG	XVI	22 751 <--	YP22 probe
AT 518	TCAATACATTACCATGAGAG	VIII	134 555 <--	junction *YHRCdelta3* / *YHRWdelta7*
AT 530	TATCACGACATGTGCGCTT	VIII	117 101 <--	junction *YHRCdelta3* / *YHRWdelta7*

Sequences of synthetic oligonucleotides used as primers for PCR amplifications to construct probes or to sequence junctions. (a) the coordinate refers to the first *CUP1* repeat unit.
